# Dietary Risks and Cardiometabolic Disease Burden Across the United States: Geographic Inequalities, Age–Period–Cohort Patterns, and Projections to 2045

**DOI:** 10.1016/j.ajpc.2026.101786

**Published:** 2026-08-07

**Authors:** Laiang Yao, Matthew J. Landry, Andrew Odegaard

**Affiliations:** a Department of Epidemiology and Biostatistics, Joe C. Wen School of Population & Public Health, University of California, Irvine, Irvine, CA, USA; b Center of Global Cardiometabolic Health and Nutrition, University of California, Irvine, Irvine, CA, USA; c Department of Global Health & Biobehavioral Sciences Joe C. Wen School of Population and Public Health, University of California, Irvine, Irvine, CA, USA

**Keywords:** Cardiometabolic diseases, Nutrition, Dietary risks, Population health

## Abstract

**Background:**

Diet and nutrition remain significant but modifiable drivers of cardiometabolic diseases (CMDs), yet the contemporary burden and future impact of diet-attributable CMDs in the United States remain incompletely characterized.

**Methods:**

We obtained data from the Global Burden of Disease (GBD) 2023. Deaths and disability-adjusted life years (DALYs) of CMDs attributable to dietary risks were estimated through a comparative risk assessment framework. Temporal patterns were assessed with estimated annual percent changes (EAPCs) and age-period-cohort models. Time-series forecasting models projected burden through 2045 under a reference scenario and a counterfactual scenario assuming a 1% annual decline in overall dietary risk exposure.

**Results:**

In 2023, dietary risks accounted for more than 300,000 deaths and approximately 8 million DALYs of CMDs in the United States. Although the age-standardized death and DALY rates of diet-attributable cardiovascular disease declined, diabetes and chronic kidney disease (CKD) followed less favorable trajectories, with CKD increasing most rapidly. Diet-associated CMD burden were disproportionately concentrated in Southern and Appalachian states, with persistent geographic inequities. Leading dietary contributors were broadly consistent across states, including low intake of fruits, vegetables, and whole grains and high intake of processed meat, sodium, and sugar-sweetened beverages. Projection analyses suggested that modest, sustained improvements in population-level diet quality could yield substantial health gains through 2045.

**Conclusion:**

Dietary risks continue to impose a substantial and uneven cardiometabolic disease burden across the United States. Divergent trends by disease and persistent geographic disparities highlight the need for targeted nutrition policies and population-wide strategies to promote healthier dietary patterns.

## Introduction

Cardiometabolic diseases (CMDs), including cardiovascular diseases (CVDs), diabetes and chronic kidney disease (CKD) remain leading contributors to premature mortality and disability in the United States (US) and worldwide[1], [2], [3]. In the U.S., CVDs accounted for more than 0.9 million deaths in 2023, and imposed substantial health-care and economic burden^[3]^. In parallel, the metabolic and renal components of CMD are highly prevalent, with recent estimates suggesting that over 50% of the population have diabetes or prediabetes, while more than 1 in 7 U.S. adults are living with CKD[4], [5]. These conditions frequently coexist and are linked by shared pathophysiological pathways. Accordingly, the Cardiovascular-Kidney-Metabolic (CKM) framework was proposed to characterize the interconnected associations among these CMDs, and promote coordinated prevention and management[6], [7].

Diet and nutrition are crucial and modifiable determinants of cardiovascular and metabolic health[8], [9]. Substantial evidence from large population-based studies has linked dietary factors and patterns to a wide range of CMDs and outcomes, and contemporary prevention frameworks increasingly recognize nutrition as central of maintaining cardiometabolic health[10], [11], [12]. The American Heart Association’s (AHA) Life’s Essential 8 identifies healthy diet as a foundational behavior for cardiovascular health, while the recently updated 2026 AHA Dietary Guidance to Improve Cardiovascular Health further emphasizes the central role of diet and nutrition in maintaining cardiometabolic health across the life course^[13]^^,^^[14]^. Similarly, the recent CKM guidance identifies diet and nutrition as central components of lifestyle modification across the CKM health continuum^[15]^.

Despite broad recognition of diet as a major prevention target, the population burden of CMDs attributable to dietary risks is unlikely to be even across the United States. Differences in food environments and the accessibility and affordability of healthy foods may contribute to persistent geographic and socioeconomic disparities in diet-related CMD burden. However, recent evidence comprehensively characterizing the national and state-level burden, long-term trends, and disparities of CMDs attributable to dietary risks remains limited. Addressing this gap is essential for identifying high-burden populations and regions and for informing targeted nutrition policies, prevention strategies, and resource allocation.

## Methods

### Data source and definitions

We obtained data for the United States overall and for each of the 50 states and the District of Columbia, among individuals aged ≥25 years, from the Global Burden of Disease Study 2023 (GBD 2023). The primary outcomes were deaths and disability-adjusted life years (DALYs) from CMDs attributable to dietary risks. GBD 2023 is an international research collaboration that provides annual estimates of disease burden for 375 causes and 88 risk factors across demographic groups from 1990 to 2023. Detailed GBD estimation and modeling procedures have been reported elsewhere^[16]^^,^^[17]^. Briefly, mortality inputs are compiled from vital registration systems, household mortality surveys, censuses, and surveillance sources and are synthesized using the Cause of Death Ensemble model (CODEm). Morbidity inputs are derived from clinical and administrative data sources, disease registries, the published literature, and population surveys, and are modeled using DisMod-MR 2.1, a Bayesian meta-regression framework.

GBD applies a hierarchical cause classification. In this study, CMD included CVDs and related cardiometabolic conditions, comprising ischemic heart disease (IHD), stroke, hypertensive heart disease, lower extremity peripheral arterial disease, atrial fibrillation and flutter, and aortic aneurysm, as well as diabetes mellitus and CKD. Analyses were stratified by sex and age group. Age-specific estimates were obtained in 5-year intervals from 25–29 through 85+. For presentation, adjacent 5-year age groups were additionally aggregated into 10-year bands (25–34, 35–44, 45–54, 55–64, 65–74, 75–84, and 85+) by calculating a weighted average of age-specific rates within each combined band using the corresponding age-group weights. Age-standardized rates (ASRs) were calculated with US standard population weights.

### Dietary risks and comparative risk assessment framework

A risk factor hierarchy of four levels was categorized by GBD 2023. In this study, we focused on dietary risks, a Level-2 behavioral risk cluster defined as suboptimal exposure to multiple dietary components. In addition to the overall dietary risks cluster, we further quantified attributable mortality and morbidity for the 12 component dietary risks: diet low in fruits, vegetables, whole grains, legumes, nuts and seeds, fiber, seafood omega-3 fatty acids, and omega-6 polyunsaturated fatty acids, and diet high in red meat, processed meat, sugar-sweetened beverages and sodium. Dietary risks refer to intakes below or above the risk-specific theoretical minimum risk exposure level (TMREL), which represents the exposure distributions associated with the lowest disease risk and were informed by observed exposure levels and risk–outcome evidence synthesized from systematic reviews and meta-analyses. The strength and consistency of each dietary risk–outcome association were further evaluated using the Burden of Proof Risk Function framework, which accounts for between-study heterogeneity and provides a conservative estimate of risk. Detailed definitions, TMRELs of each dietary risk are provided in Supplementary Table 1.

The burden of CMDs attributable to dietary risks were estimated with a comparative risk assessment (CRA) framework^[17]^. Briefly, CRA involves: (1) estimating the population exposure distribution for each dietary risk by age, sex, year, and location; (2) deriving relative risk (RR) functions for each risk–outcome pair based on pooled epidemiological evidence; (3) specifying the theoretical minimum risk exposure level (TMREL) as the counterfactual exposure associated with the lowest risk; and (4) calculating the population attributable fraction (PAF) by comparing observed exposure to the TMREL-based counterfactual distribution. For a continuous exposure, the PAF is calculated as

$$PAF=\frac{\int RR\left(x\right)P\left(x\right)dx-\int RR\left(x\right)P^{*}\left(x\right)dx}{\int RR\left(x\right)P\left(x\right)dx}$$

Where P(x) is the observed exposure distribution and $P^{*}\left(x\right)$ denotes the counterfactual distribution defined by the TMREL. For each outcome, attributable deaths and DALYs were estimated by multiplying the total outcome-specific burden by the corresponding PAF for each dietary risk. When aggregating attributable burden across multiple dietary risks, GBD applies the risk hierarchy and mediation adjustments to account for shared causal pathways and to avoid double counting, thereby ensuring internally consistent risk attribution within the GBD framework.

### Temporal trends, Geographic disparities

We assessed temporal trends in the burden of CMDs attributable to dietary risks from 1990 to 2023 using the estimated annual percent change (EAPC). For each outcome, EAPC was derived from a log-linear regression assuming a constant proportional change over time, expressed as the following formula:

$$\ln\left(ASR\right)=\alpha +\beta t+\varepsilon ,\;EAPC=100\times \left(e^{\beta }-1\right),$$

The 95% confidence interval (CI) for EAPC was calculated from the standard error of β. Trends were classified as increasing if the 95% CI was entirely >0, decreasing if entirely <0, and stable otherwise. Statistical significance was evaluated using a two-sided α=0.05.

We further compared diet-attributable CMD burden across US states and estimated Spearman correlation coefficients to assess its association with the Socio-demographic Index (SDI), a composite measure of lag-distributed income per capita, average educational attainment and total fertility rate.

We employed an age-period-cohort modelling framework to assess the independent contributions of age-effects (risk variation across life course), period effects (time-varying influences affecting all ages) and birth-cohort effects (generational differences reflecting risk exposures) of diet-associated CMD burden. Briefly, CMD deaths/DALYs attributable to dietary risks were tabulated in consecutive 5-year age groups and 5-year calendar periods, and corresponding birth cohorts were derived from the age–period structure.

### Forecasts and projections framework

We projected the total deaths and DALYs, along with corresponding ASRs of the cardiometabolic diseases attributable to dietary risks from 2024 to 2045 using time-series forecasting models. ASRs were modeled using an autoregressive integrated specification with a deterministic linear drift term, as this approach accommodates autocorrelation and non-stationarity while extending the long-term trend into the projection period. Total deaths and DALYs were modeled using an additive-error, additive-trend exponential smoothing model, which flexibly updates the underlying level and trend and is well suited to annual nonseasonal count data. Model adequacy was evaluated using residual diagnostics, including the Ljung–Box test for residual autocorrelation. Predictive performance was assessed using expanding-window, rolling-origin validation over 2014–2023, with accuracy summarized using the root mean squared error, mean absolute error, and mean absolute percentage error for 1- to 5-year forecasts. Validation results are reported in Supplementary Appendix 1.

The reference scenario extrapolated the historical summary exposure value (SEV), a scalar index of population-level dietary risk exposure linearly, while the counterfactual intervention scenario applied a 1% annual relative SEV reduction from the 2023 baseline, corresponding to an approximate 20% reduction by 2045. This prespecified scenario was intended to represent a moderate, sustained improvement achievable through population-level nutrition policies. Counterfactual outcomes were estimated by scaling the reference forecasts by the annual ratio of counterfactual to reference SEV. This approach assumes that, with an unchanged exposure–response relationship, proportional changes in aggregate SEV produce proportional changes in diet-attributable burden. Annual and cumulative differences between the reference and counterfactual scenarios were interpreted as potentially avoidable burden under the modeled assumptions. Conservative uncertainty bounds were derived from the 95% prediction intervals by pairing the lower reference bound with the upper counterfactual bound for the lower estimate and the upper reference bound with the lower counterfactual bound for the upper estimate. This approach intentionally produces wider bounds without assuming independence or specifying an unsupported covariance structure between the two scenarios. Detailed information regarding our projection models and estimation framework are provided in Supplementary Appendix 1.

## Results

In 2023, dietary risks were associated with an estimated total of 243,000 deaths (95% UI: 128-339) and 5.3 million DALYs of CVDs (95% UI: 3.0-7.1), 29,000 deaths (95% UI: 18-39) and 1.8 million DALYs of diabetes (95% UI: 1.1-2.5), along with 37,000 deaths (95% UI: 21-56) and 0.8 million DALYs of CKD (95% UI: 0.5-1.2) in the US. From 1990 to 2023, the overall numbers of deaths and DALYs from diet-attributable CMD changed modestly, with IHD, diabetes, hypertensive heart disease, and CKD consistently representing the leading contributors to mortality and morbidity across the study period (Fig. 1A–B). Diet-attributable IHD deaths and DALYs declined overall, with a relative flattening of trends after 2019. In contrast, diet-attributable burden from CKD and hypertensive heart disease increased over time, and DALYs attributable to dietary risks for diabetes also rose (Fig. 1A–B).

**Fig. 1:**
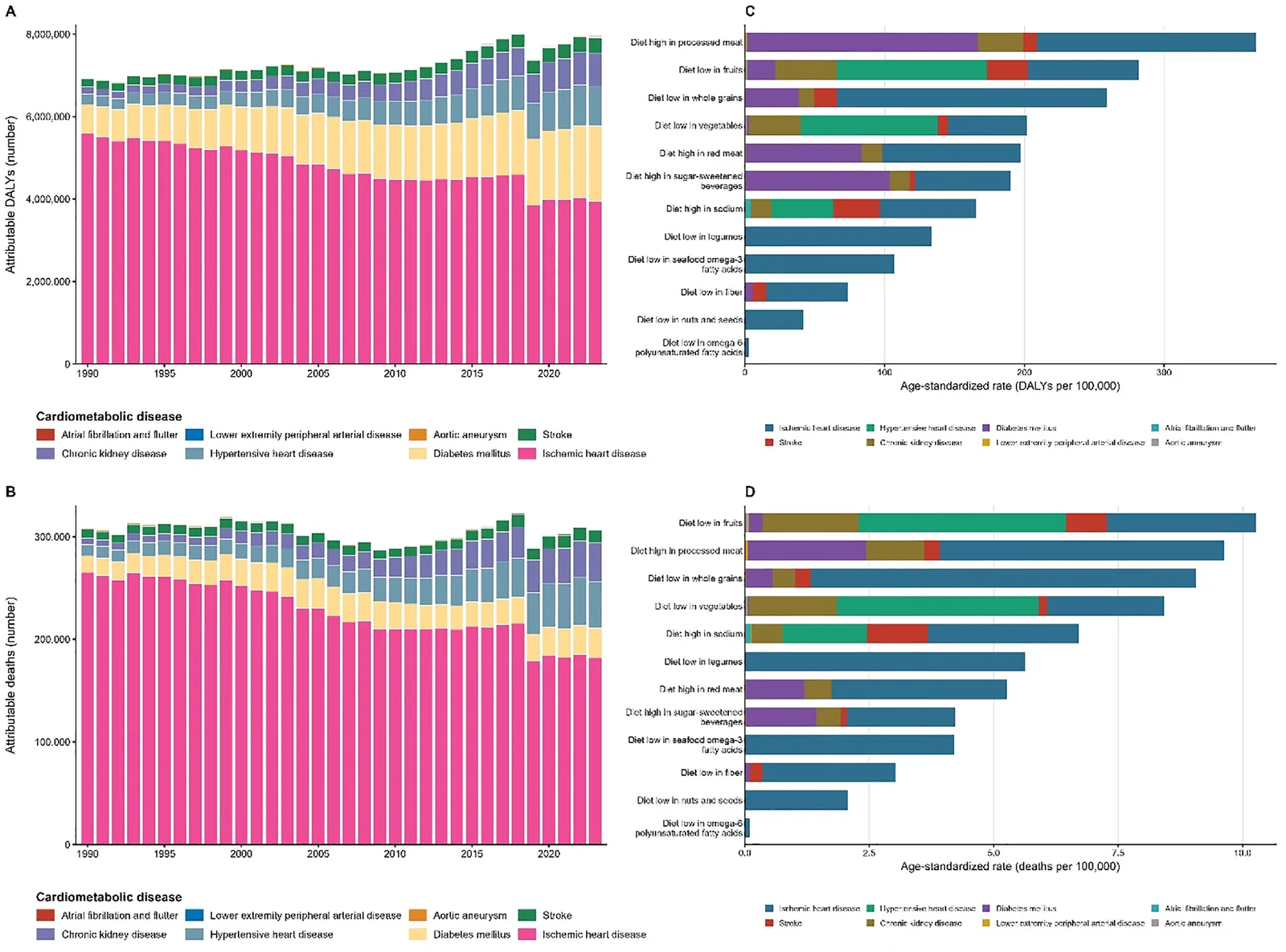
The (A) total number of deaths and (B) DALYs of cardiometabolic diseases attributable to dietary risks in the U.S. from 1990 to 2023; The (C) DALY rates and (D) death rates of cardiometabolic diseases attributable to specific dietary risks across the U.S.

In 2023, low fruit and whole-grain intake and high processed-meat intake were the leading dietary risks for diet-attributable CMD burden in the United States (Fig. 1 C-D). Dietary risk profiles varied across CMD outcomes. IHD burden was associated with all evaluated dietary components, with the largest contributions from low intakes of whole grains, legumes, and seafood omega-3 fatty acids and high processed-meat intake. Low fruit, vegetable intake and high sodium intake were the leading dietary risks for hypertensive heart disease; high processed-meat, sugar-sweetened beverage, and red-meat intake for diabetes; and low fruit and vegetable intake for CKD.

Age-specific patterns were similar across the diet-attributed CMDs, as both death and DALY rates increased sharply with age, and males consistently experienced a higher burden than females (Supplementary Figure 1). Over the three decades, the age-standardized mortality rate (ASMR) and age-standardized DALY rate (ASDR) of diet-attributable CVD declined steadily in both sexes (Fig. 2A; Supplementary Figure 2). The EAPCs for CVD were −2.48% (95% CI, −2.59 to −2.38) for ASMR and −2.14% (95% CI, −2.24 to −2.05) for ASDR, corresponding to an ASMR of 40.31 (95% UI, 22.03–55.32) and an ASDR of 990.54 (95% UI, 583.59–1300.61) per 100,000 population in 2023 (Table 1). Within CVD, hypertensive heart disease, lower extremity peripheral arterial disease, and atrial fibrillation and flutter exhibited increasing trends in both ASMR and ASDR (Table 1). In age-stratified analyses, diet-attributable death and DALY rates showed increasing rates among individuals aged 25–34 years (Fig. 2D; Supplementary Figure 3).

**Fig. 2:**
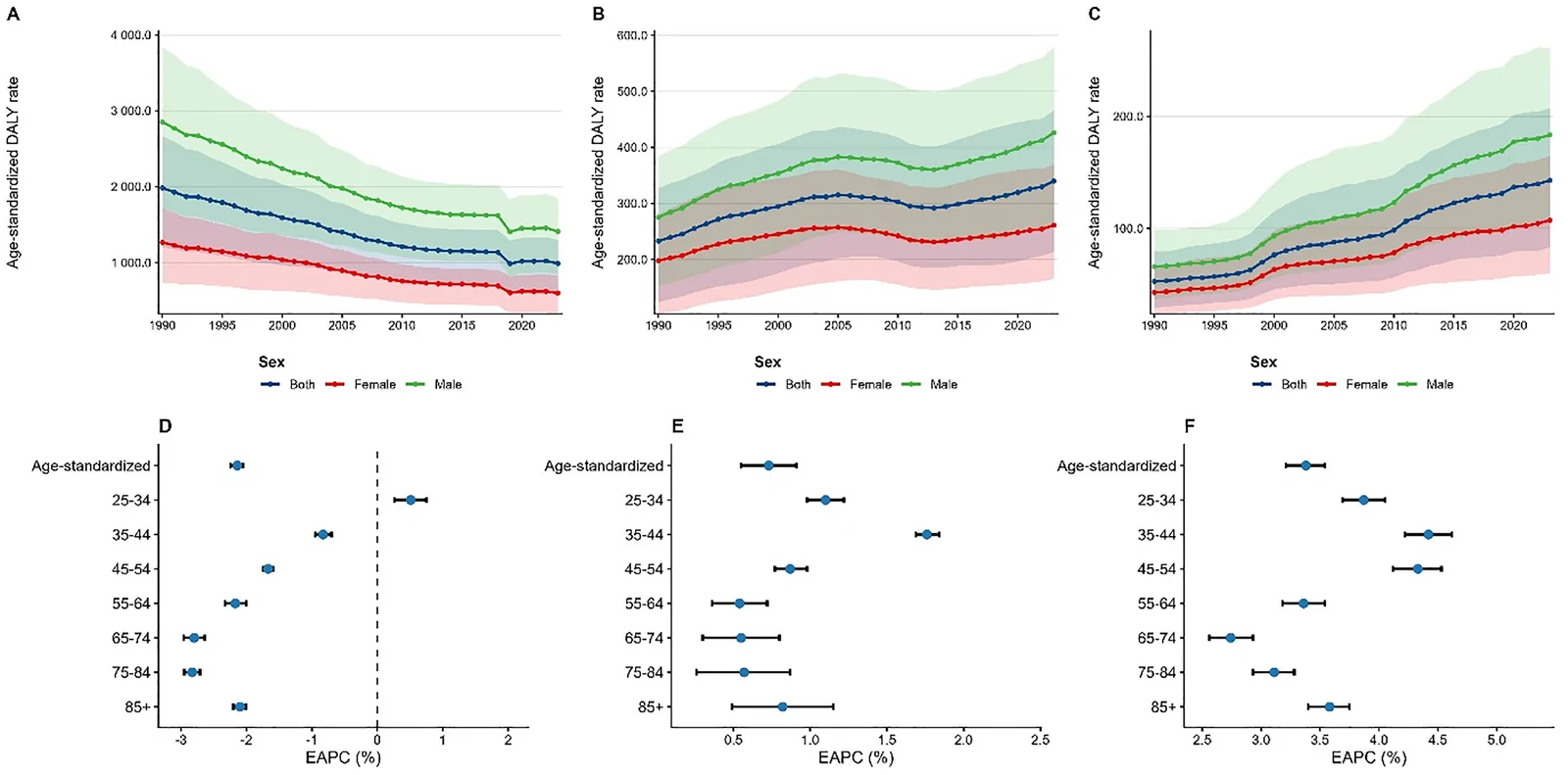
The age-standardized DALY rate of (A) cardiovascular diseases; (B) diabetes and (C) chronic kidney diseases attributable to dietary risks in the United States, from 1990 to 2023; The estimated annual percent changes in the age-standardized DALY rate of (D) cardiovascular diseases; (E) diabetes and (F) chronic kidney diseases attributable to dietary risks from 1990 to 2023 by age groups.

**Table 1:** The age-standardized mortality and DALY rates of cardiometabolic diseases attributable to dietary risks in 2023, and estimated annual percent changes from 1990-2023.

**Cardiometabolic disease**	**ASMR in 2023 (per 100k)**	**EAPC (%).**	**ASDR in 2023 (per 100k)**	**EAPC (%)**
Cardiovascular diseases	40.31 (22.03, 55.32)	-2.48 (-2.59 to -2.38)	990.54 (583.59, 1300.61)	-2.14 (-2.24 to -2.05)
Ischemic heart disease	30.24 (13.43, 45.34)	-3.1 (-3.22 to -2.99)	727.24 (358.01, 1017.99)	-2.83 (-2.93 to -2.73)
Hypertensive heart disease	7.72 (6.27, 9.46)	2.6 (2.45 to 2.76)	187.59 (156.48, 223.96)	2.71 (2.59 to 2.83)
Stroke	1.97 (-0.44, 5.46)	-0.96 (-1.2 to -0.73)	65.98 (-4.55, 157.63)	-0.05 (-0.16 to 0.06)
Lower extremity peripheral arterial disease	0.14 (0.05, 0.26)	0.72 (0.36 to 1.08)	3.22 (1.01, 5.87)	1.03 (0.73 to 1.34)
Atrial fibrillation and flutter	0.10 (0.00, 0.42)	2.25 (2.19 to 2.31)	3.26 (0.02, 13.40)	2.57 (2.36 to 2.79)
Aortic aneurysm	0.14 (0.09, 0.21)	-3.22 (-3.41 to -3.03)	3.26 (2.15, 4.82)	-2.75 (-2.94 to -2.56)
Diabetes mellitus	4.85 (3.05, 6.60)	-0.89 (-1.32 to -0.45)	339.92 (213.25, 465.90)	0.73 (0.55 to 0.91)
Chronic kidney disease	5.96 (3.44, 8.84)	3.88 (3.66 to 4.1)	142.90 (83.18, 207.44)	3.38 (3.21 to 3.54)

For diet-attributable diabetes, the ASMR increased in earlier years and then declined, with a plateau after 2012, whereas the ASDR increased throughout the period (Fig. 2B; Supplementary Figure 2). The ASDR rose by 0.73% per year (95% CI, 0.55-0.91) since 1990, reaching 339.92 per 100,000 in 2023 (Table 1). The increase in ASDR was observed across all age groups; the most rapid rises occurred in the 25–34, 35–44, 45–54, and 85+ age groups, while corresponding ASMR changes in these groups were stable (Supplementary Figure 3). For CKD, both ASMR and ASDR attributable to dietary risks increased substantially over time, with EAPCs exceeding 3% (Fig. 2C; Supplementary Figure 2). The estimates of age-standardized rates, and EAPCs for all CMDs are reported in Table 1.

Fig. 3 presents the state-level ASMRs and ASDRs of diet-attributable CMD. Overall, the burden was disproportionately concentrated in the South and parts of the Midwest, with several states in these regions showing persistently elevated rates. Diet-attributable CMDs showed significant inverse associations with SDI at the state-level, with Spearman correlations consistently around -0.7 (Supplementary Figures 4-6). In 2023, Oklahoma, Mississippi, and Arkansas had the highest CVD burden attributable to dietary risks, each exceeding 50 deaths and 1,600 DALYs per 100,000 population (Supplementary Table 2). These states also experienced comparatively slower declines over time than most other states. In contrast, Minnesota, Massachusetts, and Connecticut had the lowest age-standardized rates and demonstrated the most pronounced downward trends (Supplementary Figure 7; Supplementary Table 2).

**Fig. 3:**
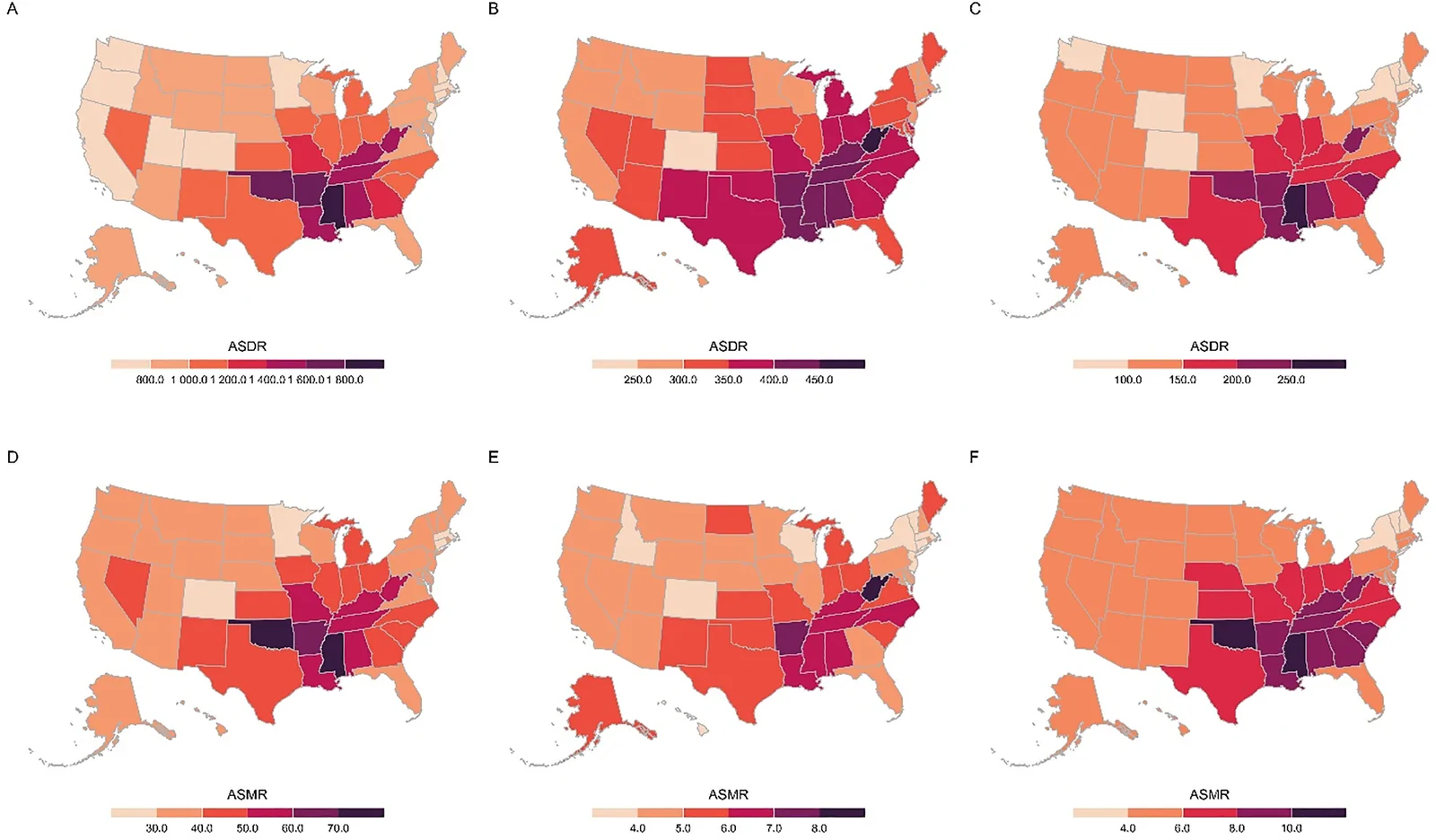
The age-standardized DALY rate of (A) cardiovascular diseases; (B) diabetes and (C) chronic kidney diseases attributable to dietary risks across the United States in 2023; the age-standardized mortality rate of (D) cardiovascular diseases; (E) diabetes and (F) chronic kidney diseases attributable to dietary risks across the United States in 2023.

State-level patterns for diabetes and CKD were broadly similar (Fig. 3). West Virginia, Arkansas, Louisiana, Kentucky, and Mississippi consistently ranked among the states with the highest ASMRs and ASDRs for both outcomes attributable to dietary risks. However, temporal trends differed more markedly for diabetes and CKD than for CVD. Several states in the Northeast exhibited relatively slower increases in diet-attributable ASRs (Supplementary Tables 3-4, Supplementary Figures 8-9). The detailed state-specific estimates of ASMRs, ASDRs, and corresponding EAPCs are provided in Supplementary Tables 2-4. The leading dietary risks were broadly consistent across states, particularly low intakes of fruits, whole grains, and vegetables and high intakes of processed meat and sodium (Supplementary Figure 10)

The APC models showed consistent age effects across the three cardiometabolic diseases, with DALY rates increasing with age and rising most sharply in later life (Fig. 4). Overall, the period and cohort effects diverged. CVD showed an overall declining period effect and a generally favorable cohort effect, whereas diabetes and CKD showed increasing period RRs and worsening cohort RRs, with particularly elevated risks in more recent cohorts (Fig. 5). The age deviations further indicated that dietary-attributable burden was consistently concentrated around ages 40-60. The local drifts indicate that individuals under 40 experienced faster increases (diabetes/CKD) or small declines (CVD) compared with older ages. The detailed APC models results were reported in Supplementary Figures 11-13.

**Fig. 4:**
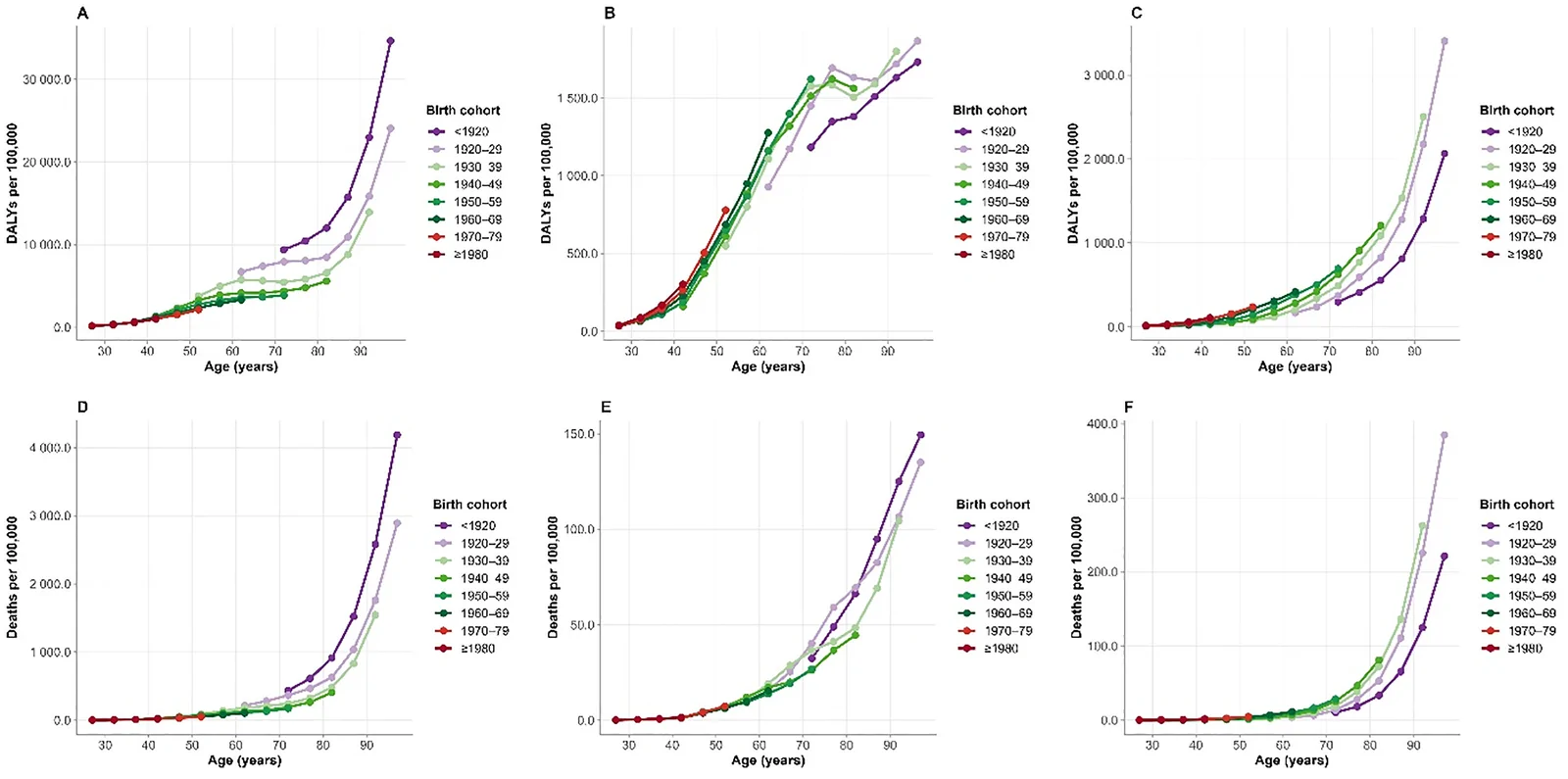
Age-specific rates of (A) CVD DALY; (B) Diabetes DALYs; (C) CKD DALYs; (D) CVD deaths; (E) Diabetes deaths; (F) CKD deaths across different birth cohorts.

**Fig. 5:**
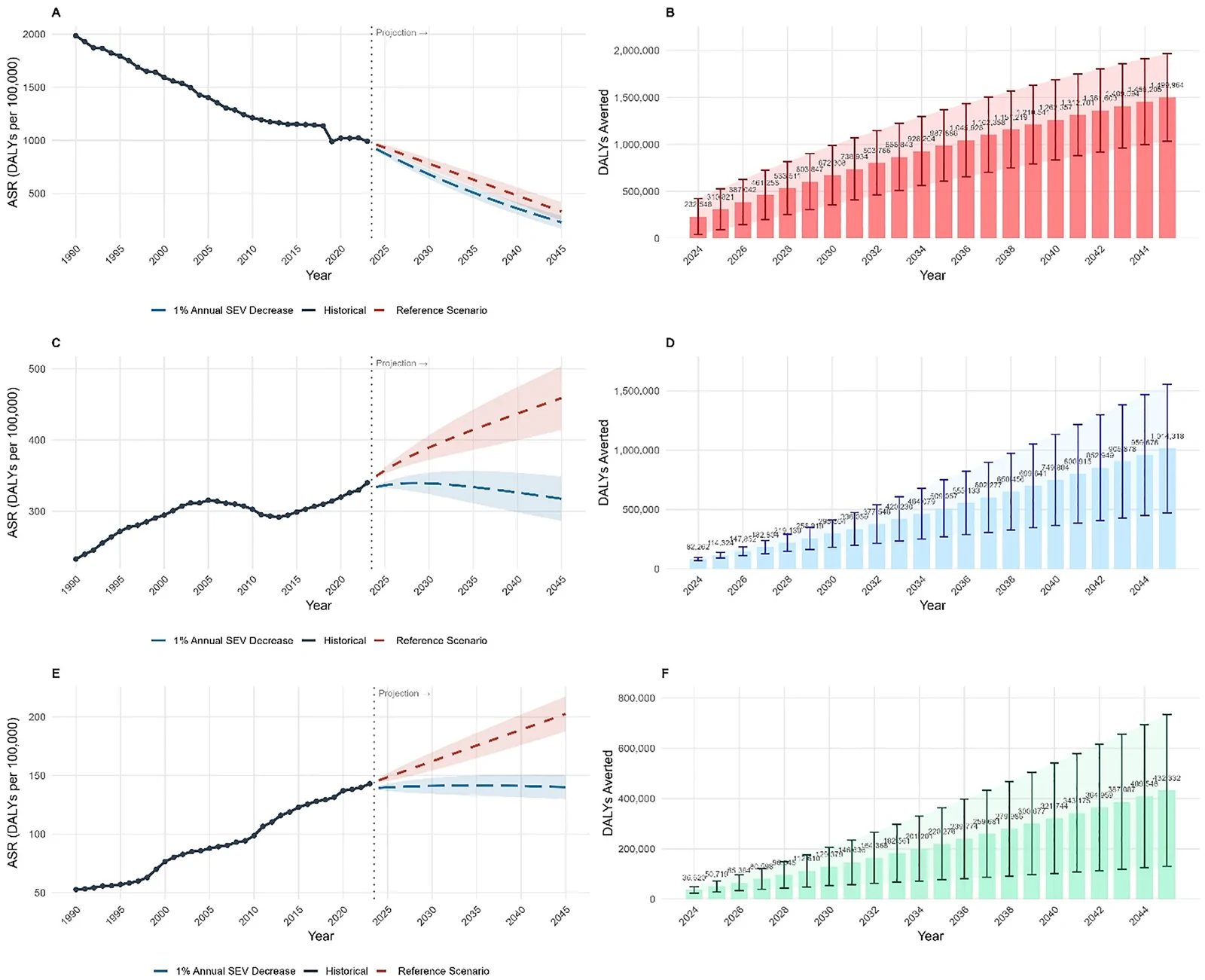
Projections and forecasts of the age-standardized DALY rate of (A) cardiovascular diseases; (C) diabetes and (E) chronic kidney diseases attributable to dietary risks in the United States till 2045 and the averted totally DALYs of (B) cardiovascular diseases; (D) diabetes and (F) chronic kidney diseases attributable to dietary risks till 2045.

We projected the diet-attributable burden of CMDs from 2024 to 2045 under a reference scenario and a counterfactual scenario assuming a 1% annual reduction in dietary risk exposures (Fig. 5). Across all CMDs, projected ASRs were lower under the counterfactual scenario. By 2045, the projected ASMRs under the counterfactual compared with the reference scenario were 5.37 (2.56–8.10) versus 7.76 (3.70–11.82) for CVD, 3.60 (2.56–4.64) versus 5.21 (3.71–6.71) for diabetes, and 5.97 (5.49–6.45) versus 8.63 (7.94–9.33) for CKD. The corresponding ASDRs were 229.12 (167.80–288.44) versus 329.87 (242.64–417.10), 317.39 (286.37–348.41) versus 458.96 (414.10–503.81), and 140.03 (129.70–150.36) versus 202.49 (187.55–217.42) per 100,000 population, respectively (Supplementary Figures 14-16 and Supplementary Tables 5–7).

Cumulatively, the differences between the two scenarios through 2045 corresponded to approximately 1.0 million fewer deaths and 20 million fewer DALYs from CVD, 140,000 fewer deaths and 11 million fewer DALYs from diabetes, and 200,000 fewer deaths and 4.8 million fewer DALYs from CKD. Detailed annual projections are provided in Supplementary Tables 5–7.

## Discussion

In this study, we systematically characterized the national and state-level burden of CMDs attributable to dietary risks in the U.S., together with their temporal patterns, demographic and geographic disparities. Despite declining rates in the burden of diet-attributable CVDs, diabetes and CKD associated with dietary risks exhibited concerning upward trajectories. Marked disparities were observed across population subgroups and regions, with a concerning rising burden among younger adults, and a disproportionate concentration in several Southern states. Across the country, the leading dietary contributors were broadly consistent, with diets high in processed meat and sodium, alongside diets low in fruits, vegetables, and whole grains accounting for a considerable share of the diet-attributable CMD burden. Importantly, our projection models suggested that a modest but sustained 1% annual decline of dietary risks exposure would yield significant population health gains. Collectively, these findings highlight dietary risks as a major yet modifiable driver of CMD burden in the US and support the need for targeted public health and nutrition policy initiatives to improve population health outcomes.

A key finding of our study was the divergent trajectories of diet-attributable CMDs. While the ASRs of diet-attributable CVDs, particularly IHD, declined, the corresponding burden for diabetes and CKD increased, with the steepest rise observed for CKD. One plausible explanation is that, over the past several decades, cardiovascular prevention and treatment have advanced rapidly and have been widely implemented at the population level[18], [19], [20]. In particular, the broad uptake of cardioprotective pharmacotherapy, particularly statins, alongside improved secondary prevention strategies, may have reduced the downstream cardiovascular consequences even when dietary risks remain prevalent^[21]^^,^^[22]^. The more favorable period and birth-cohort patterns observed for CVD were also consistent with this overall trajectory. On the other hand, the parallel rising burden of diet-related diabetes and CKD likely reflects the long-term effects of suboptimal diet, alongside the broader obesity epidemic across the U.S. Notably, the less favorable trends observed among younger adults are particularly concerning. Despite the increasing availability of therapies for diabetes and CKD, the estimated burden attributable to dietary risks remains substantial, underscoring the continued importance of diet and nutrition in preventive medicine and population health. Moreover, improvements in diet-attributable CVD burden were also heterogeneous across CVD subtypes: hypertensive heart disease and atrial fibrillation and flutter showed increasing trends despite overall CVD declines. This heterogeneity may reflect differences in underlying etiologic pathways and suggests that diet-related cardiometabolic risk is increasingly expressed through chronic, metabolic–hypertensive phenotypes rather than solely through atherosclerotic disease. Despite overall improvements with CVDs, our study highlights that metabolic and kidney outcomes will likely require earlier and more sustained prevention strategies, including upstream improvements in diet quality, to curb the growing chronic burden.

Significant and persistent population-level disparities were observed in the burden of CMDs attributable to dietary risks across the U.S. Males consistently experienced a higher diet-attributable CMD burden than females, which may reflect differences in dietary exposures, overall diet quality, health behaviors, and underlying biological susceptibility[23], [24]. Although the age-specific rates increasing markedly among higher age groups, trends were less favorable among younger adults (25–49 years), who showed faster increases or slower declines in diet-attributable CMD burden. This pattern aligns with broader evidence of rising early-onset metabolic disease and persistently high obesity prevalence among younger adults, underscoring the importance of early-life exposures and the need to prioritize preventive lifestyle interventions earlier in adulthood[25], [26]. Geographic inequities were also striking. A consistent inverse association was observed between state-level SDI and the burden of all diet-attributable CMD outcomes. Several Southern and Appalachian states had the highest burdens, together with less favorable temporal trends, indicating persistent geographic and socioeconomic inequities. Prior studies using household purchasing data showed lower nutritional quality of food purchases in Southeastern and Appalachian states and Food Acquisition and Purchase findings of the lowest diet-quality scores among participants in the South[27], [28]. Socioeconomic disadvantage may constrain healthy eating through food insecurity, the higher relative cost and limited availability of nutritious foods, inadequate transportation, time constraints, and under-resourced neighborhood food environments. These barriers are associated with poorer diet quality and may coexist with a higher prevalence of cardiometabolic risk factors and reduced access to preventive services. National evidence further indicates that improvements in diet quality have been smaller among socioeconomically disadvantaged populations and communities[29], [30], [31]. Collectively, these findings highlight the need for place-based and equity-oriented nutrition strategies that pair dietary guidance with interventions addressing the social and environmental determinants of diet quality, particularly in high-burden states.

Despite substantial state-level heterogeneity in the burden of diet-attributable CMDs, the leading dietary contributors were broadly consistent nationwide. While low intakes of fruits, vegetables, and whole grains have long been recognized as major cardiometabolic dietary risks, our findings also highlight diets high in processed meat and sugar-sweetened beverages (SSBs) as prominent drivers of CMD morbidity and mortality[32], [33]. This pattern is consistent with prospective studies and meta-analytic evidence linking processed meat and SSB consumption to higher risks of coronary heart disease, diabetes, and related cardiometabolic outcomes[34], [35], [36]. Processed meat and SSBs also reflect broader concerns about poor dietary quality and high exposure to ultra-processed foods (UPFs), which comprise more than half of total energy intake among U.S. adults and are increasingly recognized as important markers of adverse population dietary patterns^[37]^. From a public health perspective, the consistency of leading dietary risks across states suggests that core prevention targets for diet and nutrition may be nationally shared. National nutrition strategies should therefore prioritize improving overall diet quality while reducing major harmful dietary exposures, particularly those related to processed foods, added sugars, and sodium.

Our findings also align with contemporary guidance and frameworks, which places healthy dietary patterns, rather than isolated foods or nutrients, at the center of cardiovascular health promotion and disease prevention^[14]^. Established cardioprotective dietary patterns, including Mediterranean-style and DASH dietary patterns, share several features that mirror these risk factors, including greater intake of unprocessed, plant-based and fiber-rich foods, healthier sources of protein and fat, and lower consumption of processed foods, added sugars, and sodium[38], [39], [40]. Importantly, dietary quality is relevant not only to primary prevention but also to secondary prevention among individuals already living with CMDs. Emerging evidence suggests that healthier dietary patterns may improve cardiometabolic prognosis and reduce subsequent cardiovascular risk in high-risk populations, including individuals with diabetes or established cardiometabolic conditions[41], [42], [43]. Consequently, these findings reinforce the need to translate dietary guidance into both population-wide prevention strategies and clinical approaches for CMD risk management.

Our findings provide several implications for dietary and cardiometabolic health across the U.S. First, the divergent trends in CMDs support an integrated prevention approach aligned with the cardiovascular–kidney–metabolic continuum, with greater emphasis on upstream dietary improvement earlier in the life course^[15]^. Second, the persistent demographic and geographic disparities call for equity-oriented, place-based strategies. In high-burden and disadvantaged communities, priorities include strengthening the reach to nutrition programs, improving access to affordable healthy foods, and integrating food-insecurity screening, referral services, and dietary counseling into health-care and community settings. Nutrition education delivered through different settings also remains important, particularly for younger adults. Third, the consistency of leading dietary risks across states provides scalable national targets, including healthy food procurement standards and initiatives to increase fruit, vegetable, and whole-grain consumption while reducing processed meats, sugar-sweetened beverages, and sodium. Finally, our projections suggest that sustained improvements in diet quality could yield substantial health gains at the population-level, reinforcing the need for coordinated interventions across healthcare, public health, social assistance, and the broader food environment.

Several limitations should be acknowledged. First, GBD estimates are modeled and depend on the availability and quality of underlying data and modeling assumptions. Dietary exposure estimates are susceptible to measurement error and bias may remain, despite data harmonization and uncertainty propagation. Second, the comparative risk assessment framework requires assumptions regarding exposure distributions, relative risks, counterfactual specifications, mediation, and causal structure, which may influence the magnitude of attributable burden and occasionally produce counterintuitive estimates, including negative PAFs. Third, our subnational analyses were limited to the state level and did not incorporate race/ethnicity or individual level socioeconomic characteristics. Future studies with more detailed geographical levels, and incorporation of socioeconomic and racial/ethnic disparities, would provide more actionable evidence on inequities and intervention targets. Finally, our projections of future trends should be interpreted as scenario-based reference estimates rather than precise predictions. They rely on assumptions regarding the continuation of historical dietary risk exposures, proportional links between SEV and attributable burden, and a uniform annual reduction in aggregate dietary risk exposure. Future demographic changes, policy shifts, and medical advances were not explicitly incorporated and may influence future trends of CMDs.

**Figure:**
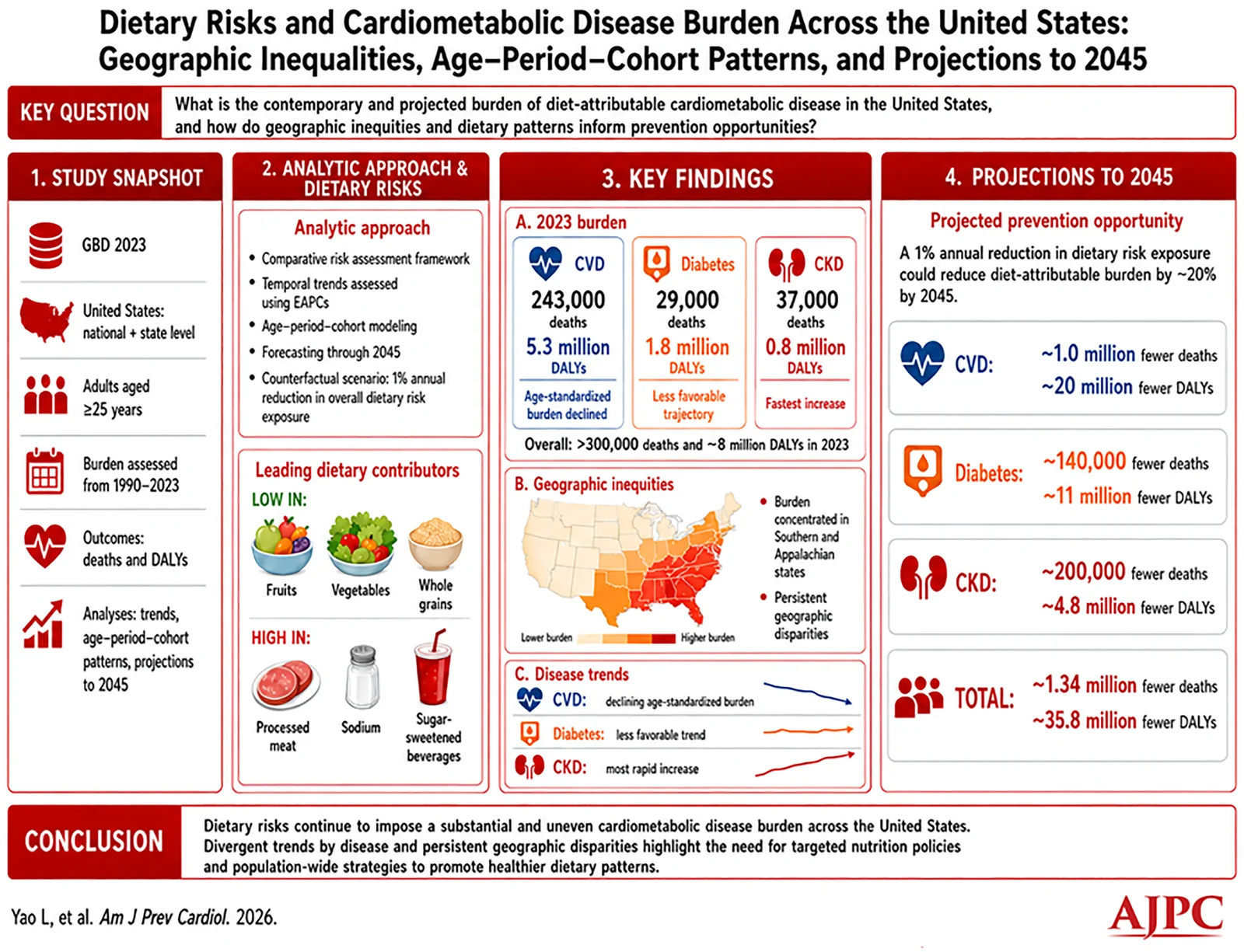
Central Illustration.

## Conclusions

In conclusion, this study provides a comprehensive assessment of the burden, trends, and disparities of cardiometabolic diseases attributable to dietary risks across the US. We identified a growing burden of diet-attributable diabetes and CKD, persistent regional disparities, and worsening epidemiological trends. Our findings further indicate that realistic improvements in dietary risk exposure could yield substantial future health benefits. These results highlight dietary risks as a major but modifiable driver of CMD burden and reinforce the importance of targeted nutrition and public health strategies to improve cardiometabolic health in the US.

## CRediT authorship contribution statement

**Laiang Yao:** Writing – review & editing, Writing – original draft, Visualization, Validation, Methodology, Investigation, Formal analysis, Data curation, Conceptualization. **Matthew J. Landry:** Writing – review & editing, Supervision, Project administration. **Andrew Odegaard:** Writing – review & editing, Validation, Supervision, Resources, Project administration, Methodology, Conceptualization.

## Declaration of competing interest

The authors declare that they have no known competing financial interests or personal relationships that could have appeared to influence the work reported in this paper.
