# Supplementary material for: Dietary Risks and Cardiometabolic Disease Burden Across the United States: Geographic Inequalities, Age–Period–Cohort Patterns, and Projections to 2045
Source: Am J Prev Cardiol. 2026 Aug 7;30:101786. doi: 10.1016/j.ajpc.2026.101786 (PMC13632084; doi:10.1016/j.ajpc.2026.101786)
Supplement: Supplementary file 1 [file mmc1.docx]

**Supplementary Appendix 1: Supplementary Methods**

**Overview of the analytical framework**

Our analytical objective was to project and forecast the future trajectory of cardiometabolic diseases, including cardiovascular diseases (CVDs), diabetes, and chronic kidney disease (CKD) attributable to aggregated dietary risks through 2045 under two pre-specified exposure scenarios, and to estimate the number of deaths that could plausibly be averted under an intervention that achieves a sustained 1% annual reduction in population-level dietary risk exposure. The framework proceeds in four sequential stages: (i) time-series models are fitted to historical age-standardised mortality rates (ASRs) and total deaths to generate reference projections; (ii) the summary exposure value (SEV) is projected under both scenarios; (iii) intervention-scenario outcomes are derived by integrating the SEV trajectories with the reference forecasts; and (iv) deaths/DALYs averted and their uncertainty are estimated by differencing the two scenario projections.

**Time-series forecasting models**

Separate time-series models were fitted to the historical ASR and total deaths series to generate reference-scenario projections through 2045. A fundamental modelling requirement was that each model must propagate the directional trend observed in the historical data into the projection horizon, rather than reverting toward the long-run mean or flattening to a constant level. Two complementary model families were therefore employed, each suited to the structural characteristics of its target series.

Historical ASRs were modelled using an autoregressive integrated moving average (ARIMA) specification augmented with a deterministic linear drift term. The general ARIMA(*p*, *d*, *q*) framework captures three structural components of a time series: the autoregressive component (AR, of order *p*) models the dependence of the current value on its own lagged values; the integrated component (I, of order *d*) removes non-stationarity through differencing; and the moving average component (MA, of order *q*) models the dependence of the current value on past forecast errors. The drift term δ is a constant added to the differenced series, introducing a deterministic linear trend in the original scale:

*ΔᵈYₜ = δ + Σ φᵢ ΔᵈYₜ₋ᵢ + Σ θⱼ εₜ₋ⱼ + εₜ*

where Δᵈ denotes the *d*-th order difference operator, φᵢ are autoregressive coefficients, θⱼ are moving average coefficients, and εₜ is a white noise error term. The drift parameter δ ensures that the secular decline in ASRs — reflecting long-run improvements in dietary quality, healthcare access, and CVD management — continues linearly into the projection period. This is critical because a drift-free integrated model would produce a random walk forecast (flat expected trajectory), which is inconsistent with the sustained downward trend observed over 1990–2023.

Model orders (*p*, *d*, *q*) were selected data-adaptively by minimzsing the Akaike Information Criterion corrected for small samples (AICc), which penalizes model complexity to guard against overfitting given the moderate sample size (*T* = 34 years). The AICc is defined as:

*AICc = −2 × logℒ(θ̂) + 2k + (2k² + 2k) / (T − k − 1)*

where logℒ(θ̂) is the maximized log-likelihood, *k* is the number of estimated parameters, and *T* is the number of observations. Parameter estimation was performed by maximum likelihood.

Total deaths and DALYs of the cardiometabolic diseases attributable to dietary risks were modelled using an exponential smoothing state-space framework with additive error, additive trend, and no seasonal component, denoted ETS(A,A,N) in standard notation. Unlike the ARIMA framework, which models autocorrelation structure in the observed series, exponential smoothing decomposes the series into unobserved state components that are updated recursively as new observations arrive. The ETS(A,A,N) model specifies:

*Yₜ = ℓₜ₋₁ + bₜ₋₁ + εₜ*

*ℓₜ = ℓₜ₋₁ + bₜ₋₁ + αεₜ*

*bₜ = bₜ₋₁ + βεₜ*

where ℓₜ is the smoothed level at time *t*, *bₜ* is the smoothed trend (slope), α ∈ (0, 1) is the level smoothing parameter, and β ∈ (0, 1) is the trend smoothing parameter. Forecasts *h* steps ahead are generated as:

*Ŷₜ₊ʰ|ₜ = ℓₜ + h × bₜ*

This specification ensures that the projected trajectory of total deaths inherits both the current level and the current slope of the series at the end of the estimation window (2023), carrying the observed trend, driven jointly by demographic growth, population ageing, and evolving dietary exposures into the future. A non-damped trend was used because the historical series exhibited consistent and sustained directionality across the full 1990–2023 window, without evidence of trend attenuation that would motivate a damped specification. Smoothing parameters (α, β) and initial state values were estimated by minimizing the sum of squared one-step-ahead forecast errors.

**Projection of Summary Exposure Values**

The summary exposure values (SEVs) is the scalar summarizing population-level prevalence of exposure to dietary risks across all severity levels, weighted by the exposure-response function. Under the reference scenario, the future SEV trajectory was estimated by fitting an ordinary least-squares (OLS) linear regression of SEV on calendar year across the full historical period (1990–2023):

*SEVₜ = α₀ + α₁ × yearₜ + εₜ*

The fitted regression coefficients (α₀, α₁) were then used to extrapolate the SEV linearly from 2024 to 2045. Projected values were constrained to the interval [0, 100] to maintain the interpretability of the SEV as a percentage. This approach assumes that the pace of change in population-level dietary risk exposure observed historically, driven by gradual shifts in food systems, agricultural production, income levels, urbanization, and dietary guidelines, continues unaltered. The reference scenario therefore represents a business-as-usual continuation of structural trends and serves as the counterfactual baseline against which the intervention scenario is evaluated.

The intervention scenario was constructed to reflect the impact of a sustained, population-wide improvement in dietary risk exposure achievable through coordinated public health action. Specifically, we modelled a compound annual 1% relative reduction in the SEV from the 2023 observed level:

*SEV*(t) = SEV₂₀₂₃ × (0.99)^(t − 2023), 2024 ≤ t ≤ 2045*

This parameterisation yields a cumulative SEV reduction of approximately 20% by 2045 relative to the 2023 baseline, as (1-0.01)²² ≈ 0.20. The choice of a 1% annual decrement reflects several converging lines of evidence and policy benchmarks. First, it is consistent with the pace of dietary improvement documented in longitudinal dietary surveillance studies in high- and middle-income countries following sustained policy engagement. Second, it aligns broadly with the ambition level of WHO’s Global Action Plan for the Prevention and Control of Noncommunicable Diseases, which calls for a 30% reduction in dietary salt intake and elimination of industrially produced trans-fatty acids by 2030, which are targets whose combined impact on aggregate dietary SEV is plausibly in the range of 1–2% annually. Third, modelling studies evaluating front-of-pack food labelling, sugar-sweetened beverage taxation, agricultural subsidy reform, and sodium reformulation initiatives individually estimate reductions of 0.3–1.5% per year in specific dietary risk metrics; a 1% aggregate annual reduction in the composite SEV is therefore a credible integrated estimate. Fourth, the compound decrement is deliberately conservative: because the 1% is applied to the prevailing SEV (not the 2023 baseline) each year, the absolute annual reduction diminishes over time, avoiding implausibly large absolute changes in later projection years.

**Integration of the SEV projections with forecasting models**

Projected ASRs and total deaths under the intervention scenario were derived by scaling the reference-scenario time-series forecasts by the ratio of the intervention SEV to the reference SEV at each projection year. This approach is grounded in the GBD comparative risk assessment (CRA) framework, in which attributable burden is a multiplicative function of the underlying background rate and the PAF, which in turn scales monotonically with the SEV for a given exposure–response function. Under the assumption of a time-invariant exposure–response relationship, a proportional reduction in the SEV produces a proportional reduction in the PAF and, consequently, in the attributable ASR and deaths.

**Estimated of averted deaths and uncertainty intervals**

The annual number of deaths averted in year *t* was calculated as the difference between the reference and intervention scenario projected deaths:

*A(t) = Deathsʳᵉᶠ(t) − Deathsᴵⁿᵗᵉʳᵛ(t)*

Cumulative deaths averted over the full projection horizon were obtained by summing *A*(*t*) across all projection years:

*Aᶜᵘᵐ = Σₜ A(t), t = 2024, …, 2045*

Uncertainty intervals (UIs) for annual and cumulative averted deaths were constructed using a conservative bound-pairing approach that simultaneously accounts for uncertainty in both the reference and intervention projections without requiring assumptions about their joint distribution or correlation structure. The lower bound of the 95% UI for averted deaths, representing the most pessimistic plausible scenario, was derived by pairing the lower prediction bound of the reference scenario with the upper prediction bound of the intervention scenario:

*Aₗₒᵂ(t) = Deathsʳᵉᶠₗₒᵂ₀₋(t) − Deathsᴵⁿᵗᵉʳᵛʰᴵᵘ₀₋(t)*

The upper bound, representing the most optimistic plausible scenario, was derived by pairing the upper prediction bound of the reference scenario with the lower prediction bound of the intervention scenario:

*Aʰᴵᵘ(t) = Deathsʳᵉᶠʰᴵᵘ₀₋(t) − Deathsᴵⁿᵗᵉʳᵛₗₒᵂ₀₋(t)*

Cumulative UI bounds were computed by summing Aₗₒᵂ(*t*) and Aʰᴵᵘ(*t*) separately across the projection horizon. This conservative pairing approach deliberately maximizes the UI width, acknowledging that the two forecast distributions may not be independent (both are conditioned on the same historical data and SEV projections). It is therefore preferable to a naïve approach that would assume independence and use the quadrature sum of individual standard errors, which could understate true uncertainty

**Supplementary Table. Validation results and model adequacy of forecasted results of cardiometabolic diseases attributable to dietary risks**

| **Cardiometabolic disease** | **Outcome** | **Ljung–Box Q (lag 6)** | **P value** | **RMSE** | **MAE** | **MAPE, %** |
| --- | --- | --- | --- | --- | --- | --- |
| Cardiovascular diseases | Mortality rate (ASMR) | 1.51 | 0.959 | 4.52 | 3.98 | 6.17 |
| Cardiovascular diseases | DALY rate (ASDR) | 0.44 | 0.979 | 54.24 | 51.74 | 8.61 |
| Diabetes mellitus | Mortality rate (ASMR) | 3.72 | 0.590 | 0.53 | 0.31 | 6.62 |
| Diabetes mellitus | DALY rate (ASDR) | 2.56 | 0.465 | 12.57 | 8.41 | 2.72 |
| Chronic kidney disease | Mortality rate (ASMR) | 3.21 | 0.686 | 0.17 | 0.12 | 2.32 |
| Chronic kidney disease | DALY rate (ASDR) | 4.16 | 0.527 | 3.76 | 2.71 | 2.12 |

**Notes.** Overall predictive-performance metrics combine 50 out-of-sample forecasts per outcome across 1- to 5-year horizons using expanding-window rolling-origin validation for target years 2014–2023.

RMSE and MAE are expressed per 100,000 population; MAPE is expressed as a percentage. The Ljung–Box test evaluates residual autocorrelation in the final model fitted to 1990–2023 data; P<0.05 indicates evidence of residual autocorrelation.

ASMR, age-standardized mortality rate; ASDR, age-standardized disability-adjusted life-year rate; RMSE, root mean squared error; MAE, mean absolute error; MAPE, mean absolute percentage error;

**Supplementary Appendix 2: Supplementary Tables and Figures**

Supplementary Table 1: Definitions of dietary risk exposures, and the theoretical minimum risk exposure level (TMREL) in the Global Burden of Disease 2023

| Dietary risk | Definition | TMREL |
| --- | --- | --- |
| Diet low in fruits | Average daily consumption (in grams per day) of fruit, including fresh, frozen, cooked, canned, or dried fruits, excluding fruit juices and salted or pickled fruits | 340-350 g/day |
| Diet low in vegetables | Average daily consumption (in grams per day) of vegetables, including fresh, frozen, cooked, canned, or dried vegetables, excluding legumes and salted or pickled vegetables, juices, nuts and seeds, and starchy vegetables such as potatoes or corn | 306-372 g/day |
| Diet low in whole grains | Average daily consumption (in grams per day) of whole grains (bran, germ, and endosperm in their natural proportion) from breakfast cereals, bread, rice, pasta, biscuits, muffins, tortillas, pancakes, and other sources | 160-210 g/day |
| Diet low in legumes | Average daily consumption (in grams per day) of legumes and pulses, including fresh, frozen, cooked, canned, or dried legumes | 100-110 g/day |
| Diet low in nuts and seeds | Average daily consumption (in grams per day) of nuts and seeds, including tree nuts and seeds and peanuts | 19-24 g/day |
| Diet low in dietary fiber | Average daily consumption (in grams per day) of fibre from all sources including fruits, vegetables, grains, legumes, and pulses | 22-25 g/day |
| Diet low in seafood omega-3 fatty acids | Average daily consumption (in milligrams per day) of eicosapentaenoic acid (EPA) and docosahexaenoic acid (DHA) | 470-660 mg/day |
| Diet low in polyunsaturated fatty acids (PUFA) | Average daily consumption (in % daily energy) from omega-6 polyunsaturated fatty acids (PUFA) (specifically linoleic acid, γ-linolenic acid, eicosadienoic acid, dihomo-γ-linolenic acid, arachidonic acid) | 9-10% of total daily energy intake |
| Diet high in processed meat | Average daily consumption (in grams per day) of meat preserved by smoking, curing, salting, or addition of chemical preservatives | 0 g/day |
| Diet high in red meat | Average daily consumption (in grams per day) of unprocessed red meat including pork and bovine meats such as beef, pork, lamb, and goat, but excluding all processed meats, poultry, fish, and eggs | 0-200 g/day |
| Diet high in sodium | Average 24 h urinary sodium measured (in grams per day) | 1-5 g/day |
| Diet high in sugar-sweetened beverages | Average daily consumption (in grams per day) of beverages with ≥50 kcal per 226.8 serving, including carbonated beverages, sodas, energy drinks, fruit drinks, but excluding 100% fruit and vegetable juices | 0 g/day |

Supplementary Table 2. The age-standardized mortality and DALY rates of cardiovascular diseases attributable to dietary risks in 2023 in the United States and states, and estimated annual percent changes from 1990-2023

| **Location** | **ASMR (per 100k)** | **EAPC (%)** | **ASDR (per 100k)** | **EAPC (%)** |
| --- | --- | --- | --- | --- |
| United States of America | 40.31 (22.03, 55.32) | -2.48***  (-2.59 to -2.38) | 990.54 (583.59, 1300.61) | -2.14***  (-2.24 to -2.05) |
| Alabama | 58.03 (33.69, 77.91) | -1.54***  (-1.63 to -1.45) | 1531.91 (943.31, 1963.16) | -1.17***  (-1.25 to -1.08) |
| Alaska | 35.28 (19.03, 48.99) | -2.31***  (-2.46 to -2.15) | 915.28 (532.52, 1213.09) | -1.97***  (-2.15 to -1.78) |
| Arizona | 37.12 (20.59, 51.27) | -2.28***  (-2.39 to -2.18) | 877.35 (520.77, 1158.63) | -2.13***  (-2.23 to -2.02) |
| Arkansas | 63.80 (35.95, 86.16) | -1.32***  (-1.45 to -1.2) | 1655.84 (987.90, 2139.94) | -0.93***  (-1.02 to -0.83) |
| California | 32.18 (16.92, 45.05) | -2.91***  (-3.03 to -2.79) | 756.92 (426.81, 1012.74) | -2.6***  (-2.71 to -2.5) |
| Colorado | 29.48 (15.93, 41.18) | -2.8***  (-2.91 to -2.68) | 685.60 (404.30, 907.82) | -2.52***  (-2.64 to -2.39) |
| Connecticut | 28.65 (15.11, 40.63) | -3.01***  (-3.18 to -2.84) | 678.40 (380.25, 914.29) | -2.76***  (-2.91 to -2.6) |
| Delaware | 39.25 (20.88, 54.12) | -2.63***  (-2.74 to -2.52) | 963.13 (564.49, 1258.48) | -2.35***  (-2.44 to -2.25) |
| District of Columbia | 46.55 (29.37, 62.91) | -1.99***  (-2.18 to -1.79) | 1190.53 (789.36, 1546.83) | -2.06***  (-2.23 to -1.89) |
| Florida | 36.09 (19.25, 49.79) | -2.58***  (-2.71 to -2.45) | 914.64 (528.40, 1205.99) | -2.11***  (-2.23 to -2) |
| Georgia | 47.39 (28.40, 63.70) | -2.19***  (-2.33 to -2.05) | 1210.27 (768.51, 1562.31) | -1.81***  (-1.94 to -1.68) |
| Hawaii | 31.03 (17.48, 42.66) | -2.1***  (-2.24 to -1.96) | 818.69 (503.56, 1074.33) | -1.65***  (-1.77 to -1.52) |
| Idaho | 36.35 (19.32, 50.47) | -2.33***  (-2.46 to -2.21) | 817.95 (469.36, 1082.54) | -2.21***  (-2.32 to -2.1) |
| Illinois | 40.92 (23.08, 55.48) | -2.72***  (-2.82 to -2.61) | 1028.99 (628.84, 1331.48) | -2.37***  (-2.46 to -2.28) |
| Indiana | 44.34 (24.04, 60.88) | -2.31***  (-2.43 to -2.19) | 1101.50 (640.88, 1440.46) | -1.9***  (-2 to -1.81) |
| Iowa | 44.97 (23.73, 61.90) | -2.19***  (-2.33 to -2.05) | 1066.31 (604.62, 1398.35) | -1.87***  (-2 to -1.74) |
| Kansas | 43.87 (23.49, 60.90) | -2.01***  (-2.17 to -1.84) | 1038.38 (599.37, 1364.14) | -1.7***  (-1.84 to -1.56) |
| Kentucky | 55.98 (30.78, 75.27) | -1.94***  (-2.06 to -1.83) | 1455.09 (859.96, 1890.18) | -1.47***  (-1.56 to -1.37) |
| Louisiana | 57.93 (33.14, 77.59) | -1.82***  (-1.92 to -1.72) | 1516.51 (933.60, 1956.66) | -1.43***  (-1.52 to -1.35) |
| Maine | 38.25 (20.47, 53.38) | -2.65***  (-2.91 to -2.4) | 882.51 (506.30, 1177.53) | -2.42***  (-2.66 to -2.18) |
| Maryland | 36.68 (20.64, 50.70) | -2.39***  (-2.49 to -2.28) | 910.82 (563.90, 1206.19) | -2.07***  (-2.16 to -1.98) |
| Massachusetts | 26.89 (13.53, 38.10) | -3.06***  (-3.17 to -2.96) | 622.21 (341.15, 841.53) | -2.93***  (-3.02 to -2.84) |
| Michigan | 47.96 (26.41, 65.21) | -2.13***  (-2.24 to -2.02) | 1160.45 (689.99, 1522.57) | -1.8***  (-1.89 to -1.71) |
| Minnesota | 26.83 (13.84, 37.92) | -3.06***  (-3.28 to -2.84) | 616.55 (346.13, 825.66) | -2.8***  (-2.99 to -2.61) |
| Mississippi | 70.59 (41.92, 93.11) | -1.64***  (-1.78 to -1.5) | 1872.63 (1168.01, 2368.16) | -1.27***  (-1.41 to -1.13) |
| Missouri | 50.61 (27.31, 68.94) | -2.13***  (-2.24 to -2.02) | 1283.98 (745.06, 1674.64) | -1.66***  (-1.75 to -1.57) |
| Montana | 38.65 (20.09, 54.27) | -1.93***  (-2.11 to -1.75) | 925.01 (514.13, 1228.73) | -1.71***  (-1.87 to -1.55) |
| Nebraska | 35.57 (18.73, 49.14) | -2.69***  (-2.88 to -2.49) | 831.32 (474.42, 1088.33) | -2.38***  (-2.56 to -2.21) |
| Nevada | 48.82 (28.62, 66.21) | -1.91***  (-2.06 to -1.77) | 1175.94 (727.87, 1518.61) | -1.74***  (-1.87 to -1.61) |
| New Hampshire | 32.85 (16.85, 47.26) | -2.86***  (-3.06 to -2.67) | 756.65 (418.57, 1022.23) | -2.65***  (-2.85 to -2.45) |
| New Jersey | 31.00 (16.37, 43.37) | -2.92***  (-3.03 to -2.81) | 727.13 (424.17, 978.23) | -2.65***  (-2.75 to -2.55) |
| New Mexico | 41.53 (21.83, 57.54) | -1.88***  (-2.04 to -1.72) | 1017.18 (589.72, 1353.95) | -1.51***  (-1.68 to -1.35) |
| New York | 38.34 (20.84, 52.77) | -2.9***  (-2.99 to -2.81) | 923.33 (545.81, 1210.57) | -2.62***  (-2.7 to -2.53) |
| North Carolina | 41.36 (22.18, 56.51) | -2.67***  (-2.81 to -2.54) | 1048.12 (604.58, 1389.92) | -2.36***  (-2.49 to -2.24) |
| North Dakota | 35.48 (18.72, 49.39) | -2.61***  (-2.73 to -2.49) | 885.94 (498.42, 1179.81) | -2.2***  (-2.32 to -2.07) |
| Ohio | 45.17 (24.00, 61.84) | -2.38***  (-2.49 to -2.27) | 1105.32 (629.36, 1469.23) | -2.02***  (-2.12 to -1.92) |
| Oklahoma | 70.71 (46.28, 91.20) | -1.26***  (-1.38 to -1.14) | 1736.28 (1171.99, 2173.07) | -0.95***  (-1.05 to -0.85) |
| Oregon | 30.60 (16.17, 43.15) | -2.99***  (-3.16 to -2.82) | 713.48 (411.35, 955.75) | -2.81***  (-2.98 to -2.64) |
| Pennsylvania | 36.86 (19.25, 51.57) | -2.77***  (-2.89 to -2.65) | 895.80 (503.54, 1202.87) | -2.45***  (-2.56 to -2.35) |
| Rhode Island | 36.24 (18.23, 50.93) | -2.78***  (-2.91 to -2.65) | 817.22 (451.53, 1103.94) | -2.64***  (-2.76 to -2.52) |
| South Carolina | 45.43 (25.99, 61.11) | -2.49***  (-2.62 to -2.36) | 1178.32 (723.67, 1530.44) | -2.13***  (-2.24 to -2.02) |
| South Dakota | 37.57 (19.82, 53.04) | -2.43***  (-2.58 to -2.27) | 928.05 (526.78, 1242.73) | -2.1***  (-2.28 to -1.92) |
| Tennessee | 58.41 (32.89, 78.03) | -1.87***  (-1.98 to -1.76) | 1478.08 (884.80, 1927.10) | -1.51***  (-1.6 to -1.42) |
| Texas | 44.21 (24.46, 60.36) | -2.3***  (-2.44 to -2.16) | 1091.04 (645.31, 1430.18) | -1.96***  (-2.09 to -1.84) |
| Utah | 33.40 (17.18, 47.02) | -2.25***  (-2.37 to -2.13) | 711.77 (402.56, 954.91) | -2.23***  (-2.34 to -2.12) |
| Vermont | 38.23 (19.84, 53.34) | -2.45***  (-2.72 to -2.18) | 869.52 (491.10, 1163.59) | -2.25***  (-2.53 to -1.96) |
| Virginia | 36.17 (19.17, 50.09) | -2.7***  (-2.83 to -2.56) | 887.07 (510.88, 1182.94) | -2.38***  (-2.51 to -2.25) |
| Washington | 33.54 (18.15, 46.55) | -2.59***  (-2.72 to -2.46) | 790.66 (462.01, 1050.77) | -2.39***  (-2.54 to -2.25) |
| West Virginia | 56.37 (30.20, 76.64) | -2.12***  (-2.27 to -1.97) | 1429.35 (829.68, 1864.09) | -1.75***  (-1.88 to -1.61) |
| Wisconsin | 36.36 (19.05, 50.64) | -2.55***  (-2.7 to -2.39) | 844.38 (480.91, 1121.70) | -2.28***  (-2.42 to -2.14) |
| Wyoming | 36.09 (18.95, 49.44) | -2.22***  (-2.32 to -2.12) | 881.35 (503.73, 1163.54) | -1.92***  (-2.01 to -1.83) |

Supplementary Table 3. The age-standardized mortality and DALY rates of diabetes attributable to dietary risks in 2023 in the United States and states, and estimated annual percent changes from 1990-2023

| **location** | **ASMR (per 100k)** | **EAPC (%)** | **ASDR (per 100k)** | **EAPC (%)** |
| --- | --- | --- | --- | --- |
| United States of America | 4.85 (3.05, 6.60) | -0.89***  (-1.32 to -0.45) | 339.92 (213.25, 465.90) | 0.73***  (0.55 to 0.91) |
| Alabama | 6.12 (3.66, 8.34) | -0.81**  (-1.38 to -0.24) | 429.52 (260.15, 590.02) | 1***  (0.75 to 1.26) |
| Alaska | 5.01 (3.15, 7.03) | -0.93***  (-1.31 to -0.56) | 309.92 (195.32, 423.05) | 0.52***  (0.35 to 0.69) |
| Arizona | 4.58 (2.88, 6.26) | -0.07  (-0.38 to 0.25) | 322.21 (203.43, 441.81) | 1.18***  (1.05 to 1.31) |
| Arkansas | 7.18 (4.31, 9.88) | 0.53*  (0.13 to 0.93) | 408.87 (250.04, 561.13) | 1.45***  (1.29 to 1.62) |
| California | 4.42 (2.83, 5.99) | -0.3  (-0.89 to 0.29) | 299.84 (188.28, 412.42) | 0.73***  (0.51 to 0.96) |
| Colorado | 3.46 (2.17, 4.71) | -1.17***  (-1.62 to -0.72) | 227.66 (143.31, 311.04) | 0.08  (-0.08 to 0.23) |
| Connecticut | 3.47 (2.21, 4.68) | -1.16***  (-1.57 to -0.74) | 281.23 (176.10, 387.00) | 0.7***  (0.51 to 0.88) |
| Delaware | 4.99 (3.03, 6.81) | -1.65***  (-2.01 to -1.29) | 380.55 (232.58, 532.05) | 0.57***  (0.45 to 0.7) |
| District of Columbia | 4.43 (2.83, 6.00) | -2.52***  (-2.98 to -2.06) | 287.50 (186.50, 394.17) | -1.12***  (-1.37 to -0.86) |
| Florida | 4.59 (2.87, 6.24) | -0.36  (-0.77 to 0.06) | 347.64 (220.09, 487.62) | 1.22***  (1.03 to 1.42) |
| Georgia | 4.99 (3.04, 6.80) | -0.49*  (-0.85 to -0.12) | 365.13 (226.43, 500.44) | 0.93***  (0.77 to 1.1) |
| Hawaii | 3.12 (1.93, 4.31) | -1.28***  (-1.72 to -0.84) | 269.54 (167.26, 374.56) | 0.68***  (0.48 to 0.89) |
| Idaho | 3.97 (2.35, 5.47) | -0.92**  (-1.42 to -0.41) | 277.46 (170.98, 382.76) | 0.55***  (0.34 to 0.76) |
| Illinois | 4.60 (2.90, 6.21) | -1.28***  (-1.72 to -0.83) | 329.83 (205.70, 452.07) | 0.51***  (0.33 to 0.7) |
| Indiana | 5.91 (3.60, 8.07) | -0.54**  (-0.9 to -0.17) | 381.43 (234.87, 524.62) | 0.91***  (0.75 to 1.07) |
| Iowa | 4.48 (2.75, 6.06) | -0.24  (-0.59 to 0.1) | 318.47 (198.74, 435.53) | 1.03***  (0.91 to 1.14) |
| Kansas | 5.14 (3.18, 6.97) | -0.41  (-0.89 to 0.06) | 348.56 (218.10, 480.04) | 1.01***  (0.83 to 1.19) |
| Kentucky | 6.62 (4.01, 8.96) | -0.41  (-0.83 to 0) | 426.50 (263.46, 580.16) | 1.04***  (0.87 to 1.21) |
| Louisiana | 6.81 (4.13, 9.25) | -1.76***  (-2.32 to -1.2) | 423.45 (259.51, 582.38) | 0.12  (-0.16 to 0.39) |
| Maine | 5.36 (3.27, 7.34) | -0.73**  (-1.15 to -0.31) | 335.08 (210.92, 467.48) | 0.69***  (0.51 to 0.87) |
| Maryland | 4.67 (2.95, 6.36) | -1.93***  (-2.39 to -1.47) | 338.33 (215.72, 465.35) | 0.35**  (0.15 to 0.55) |
| Massachusetts | 3.64 (2.32, 4.95) | -1.45***  (-1.82 to -1.07) | 257.57 (161.34, 349.18) | 0.21*  (0.01 to 0.41) |
| Michigan | 5.25 (3.28, 7.16) | -0.96***  (-1.37 to -0.55) | 352.77 (218.43, 484.27) | 0.66***  (0.49 to 0.84) |
| Minnesota | 4.00 (2.47, 5.47) | -1.19***  (-1.8 to -0.57) | 290.17 (181.17, 402.03) | 0.67***  (0.42 to 0.92) |
| Mississippi | 6.58 (3.91, 8.96) | 0.61**  (0.27 to 0.96) | 415.26 (252.37, 568.37) | 1.34***  (1.2 to 1.49) |
| Missouri | 5.20 (3.23, 7.06) | -0.81**  (-1.27 to -0.35) | 365.74 (225.19, 506.77) | 0.9***  (0.71 to 1.1) |
| Montana | 4.29 (2.58, 5.87) | -0.91***  (-1.32 to -0.51) | 264.44 (162.96, 360.70) | 0.28**  (0.11 to 0.46) |
| Nebraska | 4.68 (2.89, 6.38) | -0.35  (-0.81 to 0.11) | 322.78 (199.28, 440.32) | 0.95***  (0.78 to 1.12) |
| Nevada | 4.32 (2.64, 5.88) | -0.74**  (-1.14 to -0.33) | 342.01 (211.71, 479.06) | 1.14***  (0.99 to 1.28) |
| New Hampshire | 4.39 (2.75, 6.04) | -1.1***  (-1.54 to -0.66) | 288.56 (181.86, 394.91) | 0.6***  (0.4 to 0.8) |
| New Jersey | 3.74 (2.39, 5.07) | -2.02***  (-2.49 to -1.54) | 293.50 (184.99, 406.55) | 0.19  (-0.01 to 0.39) |
| New Mexico | 5.82 (3.46, 7.96) | -0.99***  (-1.44 to -0.54) | 369.75 (227.10, 506.65) | 0.61***  (0.41 to 0.81) |
| New York | 3.55 (2.28, 4.84) | -0.84***  (-1.22 to -0.46) | 313.82 (199.46, 435.70) | 1.12***  (0.95 to 1.29) |
| North Carolina | 6.08 (3.72, 8.29) | -0.64**  (-1.05 to -0.22) | 397.54 (239.62, 548.41) | 0.87***  (0.68 to 1.06) |
| North Dakota | 5.40 (3.34, 7.33) | -0.68**  (-1.11 to -0.24) | 346.96 (217.78, 476.27) | 0.85***  (0.7 to 1.01) |
| Ohio | 5.96 (3.74, 8.07) | -1.15***  (-1.57 to -0.73) | 390.53 (242.72, 540.13) | 0.43***  (0.27 to 0.58) |
| Oklahoma | 5.80 (3.60, 7.89) | 0.08  (-0.55 to 0.72) | 389.69 (243.36, 533.84) | 1.28***  (1 to 1.55) |
| Oregon | 4.81 (2.96, 6.52) | -0.6  (-1.21 to 0.01) | 297.23 (186.59, 408.34) | 0.61***  (0.33 to 0.89) |
| Pennsylvania | 4.77 (3.01, 6.49) | -1.11***  (-1.43 to -0.79) | 317.00 (199.48, 432.60) | 0.5***  (0.35 to 0.65) |
| Rhode Island | 4.11 (2.51, 5.67) | -1.15***  (-1.55 to -0.76) | 305.45 (188.23, 424.18) | 0.61***  (0.44 to 0.78) |
| South Carolina | 5.37 (3.35, 7.28) | -1.15***  (-1.56 to -0.73) | 385.73 (239.75, 531.42) | 0.51***  (0.35 to 0.68) |
| South Dakota | 4.78 (2.97, 6.55) | -0.48*  (-0.93 to -0.02) | 315.38 (197.47, 430.67) | 0.88***  (0.7 to 1.07) |
| Tennessee | 6.47 (3.99, 8.73) | -0.26  (-0.8 to 0.28) | 407.52 (253.23, 553.94) | 1.07***  (0.82 to 1.32) |
| Texas | 5.17 (3.23, 7.01) | -1.78***  (-2.29 to -1.27) | 379.24 (240.04, 517.22) | 0.46***  (0.25 to 0.68) |
| Utah | 4.93 (3.00, 6.73) | -1.19***  (-1.68 to -0.7) | 303.65 (188.06, 415.81) | 0.02  (-0.17 to 0.21) |
| Vermont | 3.79 (2.34, 5.16) | -1.7***  (-2.15 to -1.24) | 273.45 (170.82, 374.51) | 0.15  (-0.05 to 0.35) |
| Virginia | 5.33 (3.42, 7.23) | -0.4  (-0.85 to 0.04) | 362.91 (233.81, 492.71) | 1.11***  (0.92 to 1.3) |
| Washington | 4.33 (2.75, 5.88) | -0.93**  (-1.49 to -0.36) | 292.39 (183.70, 401.21) | 0.38***  (0.19 to 0.58) |
| West Virginia | 8.11 (4.94, 11.02) | 0.11  (-0.35 to 0.57) | 464.37 (285.82, 632.58) | 1.24***  (1.03 to 1.45) |
| Wisconsin | 3.87 (2.38, 5.32) | -1.36***  (-1.76 to -0.97) | 298.89 (184.84, 417.94) | 0.54***  (0.38 to 0.7) |
| Wyoming | 4.61 (2.86, 6.36) | -0.74**  (-1.26 to -0.22) | 278.26 (176.66, 380.23) | 0.46***  (0.24 to 0.68) |

Supplementary Table 4. The age-standardized mortality and DALY rates of chronic kidney disease attributable to dietary risks in 2023 in the United States and states, and estimated annual percent changes from 1990-2023

| **Location** | **ASMR (per 100k)** | **EAPC (%)** | **ASDR (per 100k)** | **EAPC (%)** |
| --- | --- | --- | --- | --- |
| Alabama | 9.71 (5.79, 14.15) | 3.53***  (3.27 to 3.8) | 229.75 (137.02, 329.10) | 3.5***  (3.27 to 3.73) |
| Alaska | 4.61 (2.56, 6.97) | 4.55***  (4.34 to 4.76) | 110.13 (62.38, 161.77) | 3.86***  (3.71 to 4.02) |
| Arizona | 4.34 (2.29, 6.65) | 4.04***  (3.76 to 4.33) | 111.52 (60.52, 168.20) | 3.48***  (3.29 to 3.68) |
| Arkansas | 8.99 (5.35, 12.87) | 4.37***  (4.18 to 4.56) | 216.52 (131.77, 302.33) | 4.19***  (4.02 to 4.35) |
| California | 5.25 (2.90, 7.92) | 4.31***  (4.08 to 4.55) | 132.14 (73.56, 205.14) | 3.39***  (3.22 to 3.56) |
| Colorado | 4.03 (2.19, 6.11) | 3.47***  (3.2 to 3.74) | 94.29 (50.74, 144.62) | 2.93***  (2.76 to 3.1) |
| Connecticut | 4.51 (2.46, 6.97) | 3.7***  (3.47 to 3.93) | 102.01 (54.23, 156.04) | 2.88***  (2.72 to 3.04) |
| Delaware | 5.89 (3.40, 8.79) | 3.58***  (3.39 to 3.77) | 143.41 (83.90, 208.87) | 3.25***  (3.11 to 3.4) |
| District of Columbia | 7.12 (4.03, 10.45) | 2.88***  (2.64 to 3.11) | 202.44 (115.60, 299.11) | 2.7***  (2.51 to 2.89) |
| Florida | 4.77 (2.73, 7.15) | 4.09***  (3.87 to 4.32) | 123.93 (72.13, 182.80) | 3.54***  (3.37 to 3.7) |
| Georgia | 8.20 (4.80, 12.02) | 3.59***  (3.31 to 3.87) | 199.28 (119.61, 288.52) | 3.34***  (3.12 to 3.56) |
| Hawaii | 4.66 (2.61, 7.00) | 4.11***  (3.83 to 4.4) | 131.02 (75.14, 193.59) | 3.81***  (3.62 to 4) |
| Idaho | 4.61 (2.51, 7.02) | 4.16***  (3.88 to 4.45) | 103.65 (57.78, 156.00) | 3.41***  (3.2 to 3.62) |
| Illinois | 7.10 (4.25, 10.34) | 3.58***  (3.37 to 3.79) | 167.33 (100.62, 238.34) | 3.11***  (2.96 to 3.27) |
| Indiana | 7.55 (4.37, 11.14) | 4.44***  (4.18 to 4.71) | 169.23 (100.40, 245.06) | 3.96***  (3.76 to 4.16) |
| Iowa | 5.28 (2.87, 7.94) | 4.84***  (4.61 to 5.08) | 117.46 (65.98, 174.58) | 3.81***  (3.65 to 3.98) |
| Kansas | 6.73 (3.84, 10.14) | 4.46***  (4.18 to 4.74) | 149.40 (84.65, 218.38) | 3.9***  (3.71 to 4.09) |
| Kentucky | 8.41 (4.87, 12.23) | 4.43***  (4.2 to 4.67) | 191.42 (112.34, 278.86) | 4.08***  (3.89 to 4.27) |
| Louisiana | 9.89 (5.95, 14.14) | 3.61***  (3.32 to 3.9) | 246.43 (151.76, 348.24) | 3.51***  (3.26 to 3.76) |
| Maine | 4.53 (2.43, 6.92) | 3.82***  (3.58 to 4.06) | 101.94 (56.54, 152.42) | 3.27***  (3.12 to 3.43) |
| Maryland | 5.24 (2.94, 7.61) | 2.43***  (2.28 to 2.57) | 131.25 (72.35, 193.71) | 2.28***  (2.17 to 2.39) |
| Massachusetts | 4.32 (2.38, 6.62) | 3.45***  (3.17 to 3.73) | 96.70 (54.76, 144.70) | 2.92***  (2.72 to 3.11) |
| Michigan | 5.74 (3.32, 8.39) | 3.95***  (3.7 to 4.2) | 135.46 (79.21, 198.56) | 3.34***  (3.15 to 3.52) |
| Minnesota | 4.44 (2.39, 7.05) | 3.88***  (3.56 to 4.19) | 99.35 (55.01, 149.56) | 3.17***  (2.98 to 3.36) |
| Mississippi | 11.76 (7.13, 16.61) | 4.25***  (3.97 to 4.53) | 294.48 (179.78, 411.88) | 4.17***  (3.94 to 4.4) |
| Missouri | 7.15 (4.13, 10.59) | 4.27***  (4.06 to 4.48) | 163.89 (95.09, 237.88) | 3.69***  (3.52 to 3.86) |
| Montana | 4.47 (2.46, 6.76) | 3.96***  (3.74 to 4.19) | 102.81 (58.91, 153.12) | 3.38***  (3.23 to 3.53) |
| Nebraska | 6.23 (3.48, 9.53) | 3.91***  (3.69 to 4.13) | 134.15 (76.23, 194.04) | 3.3***  (3.13 to 3.46) |
| Nevada | 5.78 (3.36, 8.53) | 3.2***  (2.76 to 3.65) | 141.32 (79.77, 204.37) | 3.01***  (2.7 to 3.32) |
| New Hampshire | 3.85 (2.13, 6.07) | 4.01***  (3.77 to 4.26) | 85.58 (46.24, 135.29) | 3.13***  (2.97 to 3.29) |
| New Jersey | 4.55 (2.52, 6.81) | 3.06***  (2.8 to 3.32) | 107.31 (61.10, 159.54) | 2.53***  (2.32 to 2.74) |
| New Mexico | 5.15 (2.80, 7.79) | 3.98***  (3.76 to 4.2) | 133.73 (74.20, 198.91) | 3.72***  (3.58 to 3.87) |
| New York | 3.61 (2.03, 5.50) | 2.92***  (2.63 to 3.22) | 93.59 (52.57, 142.14) | 2.18***  (1.98 to 2.38) |
| North Carolina | 7.50 (4.36, 11.17) | 3.72***  (3.53 to 3.91) | 177.25 (106.40, 260.79) | 3.38***  (3.23 to 3.53) |
| North Dakota | 5.32 (2.95, 7.77) | 4.21***  (3.99 to 4.44) | 122.64 (69.31, 180.43) | 3.61***  (3.46 to 3.76) |
| Ohio | 6.30 (3.49, 9.38) | 3.98***  (3.75 to 4.21) | 145.91 (80.54, 216.40) | 3.56***  (3.38 to 3.74) |
| Oklahoma | 10.41 (6.10, 15.17) | 5.17***  (4.96 to 5.37) | 232.70 (139.86, 339.61) | 4.7***  (4.53 to 4.87) |
| Oregon | 4.98 (2.73, 7.72) | 4.23***  (3.98 to 4.48) | 112.90 (61.82, 168.10) | 3.51***  (3.34 to 3.67) |
| Pennsylvania | 5.60 (3.18, 8.47) | 3.56***  (3.35 to 3.78) | 127.61 (72.07, 189.45) | 3.05***  (2.87 to 3.23) |
| Rhode Island | 4.16 (2.35, 6.36) | 3.89***  (3.63 to 4.14) | 96.27 (53.83, 144.72) | 3.05***  (2.86 to 3.24) |
| South Carolina | 8.51 (5.04, 12.33) | 3.55***  (3.37 to 3.73) | 202.99 (120.75, 289.35) | 3.28***  (3.13 to 3.44) |
| South Dakota | 4.74 (2.61, 7.29) | 4.26***  (4.05 to 4.47) | 109.66 (62.23, 163.33) | 3.51***  (3.36 to 3.66) |
| Tennessee | 8.48 (4.95, 11.93) | 4.47***  (4.29 to 4.64) | 196.74 (116.03, 280.82) | 3.96***  (3.81 to 4.11) |
| Texas | 7.09 (4.15, 10.47) | 4.37***  (4.1 to 4.64) | 168.20 (97.10, 247.03) | 4.03***  (3.81 to 4.26) |
| United States of America | 5.96 (3.44, 8.84) | 3.88***  (3.66 to 4.1) | 142.90 (83.18, 207.44) | 3.38***  (3.21 to 3.54) |
| Utah | 5.46 (2.83, 8.45) | 4.48***  (4.21 to 4.76) | 111.91 (61.00, 169.76) | 3.63***  (3.42 to 3.83) |
| Vermont | 3.92 (2.25, 5.89) | 3.51***  (3.27 to 3.75) | 88.44 (50.12, 132.07) | 2.85***  (2.7 to 3) |
| Virginia | 6.43 (3.77, 9.30) | 3.36***  (3.19 to 3.53) | 148.96 (86.91, 214.94) | 3***  (2.86 to 3.13) |
| Washington | 4.07 (2.25, 6.26) | 3.92***  (3.68 to 4.16) | 98.28 (54.09, 151.42) | 3.17***  (3.01 to 3.33) |
| West Virginia | 8.69 (5.02, 12.71) | 4.81***  (4.55 to 5.07) | 201.01 (120.19, 288.52) | 4.52***  (4.32 to 4.73) |
| Wisconsin | 4.87 (2.71, 7.47) | 4.11***  (3.81 to 4.41) | 111.65 (61.90, 167.13) | 3.22***  (3.03 to 3.41) |
| Wyoming | 4.12 (2.17, 6.41) | 4.65***  (4.39 to 4.92) | 94.88 (52.18, 143.61) | 3.76***  (3.58 to 3.95) |

Supplementary Table 5. The projected and forecasted deaths and DALYs of CVDs attributable to dietary risks, with averted burden till 2045

|  | **Deaths** | | | | **DALYs** | | | |
| --- | --- | --- | --- | --- | --- | --- | --- | --- |
| **Year** | **ASR reference** | **ASR reduction** | **Averted deaths** | **Cumulative averted deaths** | **ASR reference** | **ASR reduction** | **Averted DALYs** | **Cumulative averted DALYs** |
| 2024 | 38.82 (37.85, 39.79) | 37.13 (36.20, 38.05) | 10593 (481, 20706) | 10593 (481, 20706) | 960.95 (939.91, 981.99) | 919.01 (898.89, 939.13) | 232548 (42249, 422848) | 232548 (42249, 422848) |
| 2025 | 37.34 (36.06, 38.63) | 35.15 (33.94, 36.37) | 14184 (1826, 26541) | 24777 (2307, 47247) | 930.83 (903.06, 958.61) | 876.31 (850.16, 902.46) | 310821 (92983, 528659) | 543369 (135232, 951507) |
| 2026 | 35.86 (34.31, 37.41) | 33.24 (31.80, 34.67) | 17693 (3463, 31923) | 42470 (5770, 79170) | 900.79 (867.40, 934.19) | 834.81 (803.87, 865.76) | 387042 (145472, 628612) | 930411 (280704, 1580119) |
| 2027 | 34.38 (32.61, 36.16) | 31.37 (29.75, 32.99) | 21123 (5261, 36985) | 63593 (11031, 116155) | 870.74 (832.58, 908.91) | 794.42 (759.60, 829.24) | 461258 (198705, 723810) | 1391669 (479409, 2303929) |
| 2028 | 32.91 (30.94, 34.88) | 29.55 (27.79, 31.32) | 24475 (7151, 41798) | 88067 (18182, 157953) | 840.70 (798.29, 883.10) | 755.10 (717.01, 793.19) | 533511 (252087, 814936) | 1925180 (731496, 3118865) |
| 2029 | 31.43 (29.28, 33.58) | 27.79 (25.89, 29.69) | 27751 (9096, 46406) | 115818 (27278, 204359) | 810.65 (764.38, 856.91) | 716.83 (675.92, 757.74) | 603847 (305243, 902452) | 2529028 (1036738, 4021317) |
| 2030 | 29.95 (27.63, 32.26) | 26.07 (24.06, 28.09) | 30953 (11070, 50836) | 146771 (38348, 255194) | 780.60 (730.78, 830.42) | 679.59 (636.21, 722.96) | 672308 (357919, 986697) | 3201335 (1394657, 5008014) |
| 2031 | 28.47 (26.00, 30.94) | 24.40 (22.28, 26.52) | 34082 (13056, 55108) | 180853 (51404, 310302) | 750.55 (697.41, 803.69) | 643.34 (597.79, 688.89) | 738934 (409939, 1067929) | 3940269 (1804596, 6075943) |
| 2032 | 26.99 (24.37, 29.61) | 22.78 (20.57, 24.99) | 37140 (15042, 59238) | 217993 (66446, 369540) | 720.50 (664.24, 776.76) | 608.08 (560.59, 655.56) | 803766 (461177, 1146354) | 4744035 (2265773, 7222297) |
| 2033 | 25.51 (22.76, 28.27) | 21.20 (18.91, 23.49) | 40128 (17018, 63238) | 258121 (83464, 432778) | 690.45 (631.23, 749.68) | 573.76 (524.55, 622.97) | 866843 (511544, 1222143) | 5610878 (2777317, 8444440) |
| 2034 | 24.03 (21.15, 26.92) | 19.66 (17.30, 22.03) | 43048 (18979, 67117) | 301169 (102443, 499895) | 660.40 (598.36, 722.45) | 540.37 (489.60, 591.13) | 928204 (560972, 1295437) | 6539083 (3338289, 9739877) |
| 2035 | 22.55 (19.54, 25.56) | 18.17 (15.74, 20.60) | 45901 (20919, 70883) | 347070 (123363, 570778) | 630.36 (565.62, 695.09) | 507.88 (455.72, 560.04) | 987886 (609412, 1366361) | 7526969 (3947701, 11106238) |
| 2036 | 21.07 (17.94, 24.21) | 16.72 (14.23, 19.21) | 48689 (22835, 74543) | 395760 (146198, 645321) | 600.31 (532.98, 667.63) | 476.28 (422.86, 529.70) | 1045926 (656831, 1435021) | 8572895 (4604531, 12541259) |
| 2037 | 19.59 (16.35, 22.84) | 15.31 (12.77, 17.85) | 51413 (24724, 78103) | 447173 (170921, 723424) | 570.26 (500.44, 640.08) | 445.54 (390.99, 500.08) | 1102358 (703203, 1501514) | 9675253 (5307734, 14042773) |
| 2038 | 18.12 (14.76, 21.48) | 13.94 (11.35, 16.52) | 54074 (26582, 81567) | 501247 (197503, 804991) | 540.21 (467.99, 612.43) | 415.63 (360.06, 471.20) | 1157219 (748511, 1565926) | 10832472 (6056245, 15608699) |
| 2039 | 16.64 (13.17, 20.11) | 12.61 (9.98, 15.23) | 56674 (28409, 84940) | 557921 (225912, 889930) | 510.16 (435.61, 584.71) | 386.55 (330.06, 443.03) | 1210541 (792746, 1628335) | 12043013 (6848991, 17237035) |
| 2040 | 15.16 (11.58, 18.73) | 11.31 (8.64, 13.98) | 59214 (30202, 88226) | 617135 (256114, 978156) | 480.11 (403.30, 556.92) | 358.26 (300.94, 415.57) | 1262357 (835901, 1688813) | 13305370 (7684892, 18925848) |
| 2041 | 13.68 (10.00, 17.36) | 10.05 (7.35, 12.76) | 61695 (31962, 91428) | 678830 (288076, 1069584) | 450.06 (371.06, 529.07) | 330.75 (272.69, 388.81) | 1312701 (877976, 1747426) | 14618071 (8562868, 20673274) |
| 2042 | 12.20 (8.42, 15.98) | 8.83 (6.10, 11.56) | 64118 (33686, 94550) | 742949 (321763, 1164134) | 420.01 (338.88, 501.15) | 304.00 (245.27, 362.73) | 1361603 (918972, 1804234) | 15979674 (9481840, 22477508) |
| 2043 | 10.72 (6.85, 14.59) | 7.64 (4.88, 10.40) | 66485 (35375, 97595) | 809434 (357138, 1261730) | 389.97 (306.75, 473.18) | 277.99 (218.67, 337.31) | 1409094 (958892, 1859297) | 17388768 (10440732, 24336805) |
| 2044 | 9.24 (5.27, 13.21) | 6.49 (3.70, 9.28) | 68797 (37028, 100566) | 878231 (394167, 1362296) | 359.92 (274.67, 445.16) | 252.70 (192.85, 312.56) | 1455205 (997743, 1912666) | 18843973 (11438475, 26249471) |
| 2045 | 7.76 (3.70, 11.82) | 5.37 (2.56, 8.18) | 71055 (38645, 103464) | 949286 (432812, 1465760) | 329.87 (242.64, 417.10) | 228.12 (167.80, 288.44) | 1499964 (1035534, 1964394) | 20343937 (12474009, 28213865) |

Supplementary Table 6. The projected and forecasted deaths and DALYs of diabetes attributable to dietary risks, with averted burden till 2045.

|  | **Deaths** | | | | **DALYs** | | | |
| --- | --- | --- | --- | --- | --- | --- | --- | --- |
| **Year** | **ASR reference** | **ASR reduction** | **Averted deaths** | **Cumulative averted deaths** | **ASR reference** | **ASR reduction** | **Averted DALYs** | **Cumulative averted DALYs** |
| 2024 | 4.87 (4.80, 4.95) | 4.66 (4.59, 4.73) | 1275 (866, 1685) | 1275 (866, 1685) | 349.09 (346.84, 351.34) | 333.85 (331.71, 336.00) | 82262 (69923, 94600) | 82262 (69923, 94600) |
| 2025 | 4.89 (4.74, 5.04) | 4.61 (4.46, 4.75) | 1736 (961, 2510) | 3011 (1827, 4195) | 357.29 (352.67, 361.92) | 336.37 (332.01, 340.72) | 114324 (90705, 137944) | 196586 (160628, 232544) |
| 2026 | 4.91 (4.68, 5.15) | 4.55 (4.33, 4.77) | 2200 (952, 3447) | 5211 (2779, 7643) | 364.72 (357.54, 371.90) | 338.01 (331.36, 344.66) | 147852 (109727, 185978) | 344438 (270355, 418522) |
| 2027 | 4.93 (4.61, 5.25) | 4.50 (4.20, 4.79) | 2668 (873, 4463) | 7879 (3652, 12106) | 371.53 (361.75, 381.31) | 338.96 (330.04, 347.89) | 182804 (127894, 237715) | 527243 (398249, 656237) |
| 2028 | 4.95 (4.54, 5.35) | 4.44 (4.08, 4.81) | 3140 (737, 5544) | 11019 (4389, 17650) | 377.84 (365.48, 390.21) | 339.37 (328.26, 350.48) | 219139 (145600, 292679) | 746382 (543848, 948916) |
| 2029 | 4.96 (4.47, 5.45) | 4.39 (3.96, 4.82) | 3616 (553, 6679) | 14635 (4941, 24329) | 383.75 (368.86, 398.65) | 339.34 (326.17, 352.51) | 256819 (163076, 350562) | 1003201 (706925, 1299477) |
| 2030 | 4.98 (4.41, 5.55) | 4.34 (3.84, 4.83) | 4096 (327, 7864) | 18731 (5269, 32193) | 389.34 (371.99, 406.70) | 338.96 (323.86, 354.07) | 295804 (180478, 411130) | 1299005 (887403, 1710608) |
| 2031 | 5.00 (4.34, 5.65) | 4.28 (3.72, 4.84) | 4579 (65, 9092) | 23310 (5334, 41285) | 394.68 (374.96, 414.40) | 338.31 (321.40, 355.21) | 336059 (197918, 474199) | 1635064 (1085321, 2184807) |
| 2032 | 5.01 (4.28, 5.74) | 4.23 (3.62, 4.84) | 5065 (-230, 10360) | 28375 (5104, 51645) | 399.81 (377.81, 421.81) | 337.43 (318.86, 356.00) | 377546 (215481, 539610) | 2012610 (1300803, 2724417) |
| 2033 | 5.03 (4.23, 5.83) | 4.18 (3.51, 4.84) | 5554 (-555, 11664) | 33929 (4549, 63309) | 404.78 (380.59, 428.97) | 336.37 (316.27, 356.47) | 420230 (233233, 607228) | 2432840 (1534035, 3331645) |
| 2034 | 5.04 (4.17, 5.92) | 4.13 (3.41, 4.84) | 6047 (-907, 13001) | 39976 (3642, 76310) | 409.62 (383.32, 435.91) | 335.17 (313.65, 356.68) | 464079 (251224, 676933) | 2896918 (1785259, 4008578) |
| 2035 | 5.06 (4.12, 6.00) | 4.08 (3.32, 4.83) | 6542 (-1284, 14369) | 46518 (2357, 90679) | 414.35 (386.04, 442.66) | 333.85 (311.04, 356.66) | 509057 (269496, 748618) | 3405975 (2054754, 4757196) |
| 2036 | 5.07 (4.07, 6.08) | 4.03 (3.23, 4.82) | 7041 (-1684, 15765) | 53559 (673, 106444) | 419.00 (388.75, 449.25) | 332.43 (308.43, 356.43) | 555133 (288080, 822187) | 3961109 (2342835, 5579383) |
| 2037 | 5.09 (4.02, 6.16) | 3.98 (3.14, 4.81) | 7541 (-2105, 17188) | 61100 (-1432, 123632) | 423.58 (391.47, 455.69) | 330.94 (305.85, 356.03) | 602277 (307004, 897549) | 4563385 (2649838, 6476932) |
| 2038 | 5.10 (3.97, 6.23) | 3.93 (3.06, 4.80) | 8045 (-2546, 18636) | 69145 (-3978, 142268) | 428.10 (394.20, 462.01) | 329.38 (303.29, 355.47) | 650456 (326286, 974625) | 5213841 (2976124, 7451558) |
| 2039 | 5.12 (3.93, 6.31) | 3.88 (2.98, 4.78) | 8551 (-3004, 20106) | 77696 (-6982, 162374) | 432.59 (396.96, 468.22) | 327.77 (300.77, 354.77) | 699641 (345943, 1053339) | 5913482 (3322067, 8504897) |
| 2040 | 5.13 (3.89, 6.38) | 3.83 (2.90, 4.76) | 9059 (-3480, 21599) | 86756 (-10461, 183973) | 437.03 (399.74, 474.33) | 326.11 (298.28, 353.95) | 749804 (365986, 1133621) | 6663285 (3688053, 9638518) |
| 2041 | 5.15 (3.85, 6.45) | 3.78 (2.83, 4.74) | 9570 (-3971, 23111) | 96326 (-14433, 207084) | 441.46 (402.55, 480.36) | 324.42 (295.83, 353.02) | 800915 (386425, 1215406) | 7464201 (4074478, 10853924) |
| 2042 | 5.16 (3.81, 6.52) | 3.74 (2.76, 4.72) | 10083 (-4477, 24643) | 106409 (-18910, 231727) | 445.85 (405.39, 486.32) | 322.70 (293.41, 351.99) | 852949 (407265, 1298633) | 8317150 (4481743, 12152558) |
| 2043 | 5.18 (3.78, 6.58) | 3.69 (2.69, 4.69) | 10598 (-4997, 26193) | 117007 (-23907, 257921) | 450.23 (408.26, 492.21) | 320.95 (291.03, 350.87) | 905878 (428511, 1383245) | 9223028 (4910253, 13535803) |
| 2044 | 5.19 (3.74, 6.65) | 3.65 (2.63, 4.67) | 11115 (-5531, 27760) | 128122 (-29438, 285681) | 454.60 (411.17, 498.03) | 319.18 (288.69, 349.68) | 959676 (450164, 1469188) | 10182704 (5360418, 15004991) |
| 2045 | 5.21 (3.71, 6.71) | 3.60 (2.56, 4.64) | 11634 (-6076, 29344) | 139755 (-35514, 315025) | 458.96 (414.10, 503.81) | 317.39 (286.37, 348.41) | 1014318 (472226, 1556411) | 11197023 (5832643, 16561402) |

Supplementary Table 7. The projected and forecasted deaths and DALYs of CKD attributable to dietary risks, with averted burden till 2045

|  | **Deaths** | | | | **DALYs** | | | |
| --- | --- | --- | --- | --- | --- | --- | --- | --- |
| **Year** | **ASR reference** | **ASR reduction** | **Averted deaths** | **Cumulative averted deaths** | **ASR reference** | **ASR reduction** | **Averted DALYs** | **Cumulative averted DALYs** |
| 2024 | 6.08 (5.99, 6.17) | 5.81 (5.73, 5.90) | 1696 (973, 2419) | 1696 (973, 2419) | 145.76 (143.89, 147.63) | 139.40 (137.61, 141.18) | 36620 (23874, 49365) | 36620 (23874, 49365) |
| 2025 | 6.20 (6.04, 6.36) | 5.84 (5.69, 5.98) | 2357 (1208, 3506) | 4053 (2180, 5926) | 148.52 (145.26, 151.78) | 139.82 (136.75, 142.89) | 50719 (28909, 72529) | 87339 (52783, 121895) |
| 2026 | 6.32 (6.11, 6.53) | 5.86 (5.66, 6.05) | 3048 (1474, 4622) | 7101 (3654, 10548) | 151.25 (146.81, 155.69) | 140.17 (136.05, 144.29) | 65384 (34006, 96762) | 152723 (86789, 218656) |
| 2027 | 6.44 (6.18, 6.70) | 5.88 (5.64, 6.11) | 3768 (1755, 5781) | 10869 (5409, 16329) | 153.96 (148.50, 159.42) | 140.46 (135.48, 145.44) | 80598 (38990, 122205) | 233320 (125779, 340862) |
| 2028 | 6.56 (6.26, 6.86) | 5.90 (5.63, 6.17) | 4517 (2047, 6987) | 15386 (7456, 23316) | 156.66 (150.32, 163.00) | 140.71 (135.01, 146.41) | 96345 (43856, 148833) | 329665 (169635, 489695) |
| 2029 | 6.69 (6.35, 7.02) | 5.91 (5.62, 6.21) | 5293 (2349, 8237) | 20679 (9806, 31553) | 159.36 (152.23, 166.49) | 140.91 (134.61, 147.22) | 112610 (48631, 176589) | 442275 (218267, 666284) |
| 2030 | 6.81 (6.44, 7.18) | 5.93 (5.61, 6.25) | 6097 (2661, 9533) | 26776 (12467, 41086) | 162.05 (154.21, 169.90) | 141.08 (134.25, 147.91) | 129379 (53348, 205410) | 571654 (271614, 871694) |
| 2031 | 6.93 (6.53, 7.33) | 5.94 (5.60, 6.28) | 6926 (2981, 10871) | 33702 (15448, 51956) | 164.75 (156.25, 173.25) | 141.22 (133.93, 148.51) | 146636 (58034, 235237) | 718290 (329649, 1106931) |
| 2032 | 7.05 (6.62, 7.48) | 5.95 (5.59, 6.31) | 7781 (3312, 12250) | 41483 (18760, 64206) | 167.44 (158.33, 176.56) | 141.32 (133.63, 149.01) | 164368 (62719, 266017) | 882658 (392367, 1372948) |
| 2033 | 7.17 (6.72, 7.63) | 5.96 (5.58, 6.34) | 8660 (3652, 13668) | 50143 (22412, 77874) | 170.14 (160.45, 179.83) | 141.38 (133.34, 149.43) | 182561 (67423, 297698) | 1065218 (459791, 1670646) |
| 2034 | 7.29 (6.82, 7.77) | 5.97 (5.58, 6.36) | 9563 (4002, 15124) | 59706 (26414, 92998) | 172.84 (162.61, 183.06) | 141.42 (133.05, 149.79) | 201201 (72167, 330235) | 1266419 (531958, 2000881) |
| 2035 | 7.42 (6.91, 7.92) | 5.98 (5.57, 6.38) | 10490 (4363, 16617) | 70196 (30777, 109615) | 175.53 (164.79, 186.27) | 141.43 (132.77, 150.08) | 220276 (76967, 363586) | 1486695 (608925, 2364466) |
| 2036 | 7.54 (7.01, 8.06) | 5.98 (5.56, 6.40) | 11439 (4734, 18144) | 81635 (35511, 127759) | 178.23 (167.00, 189.46) | 141.40 (132.49, 150.31) | 239774 (81836, 397711) | 1726469 (690761, 2762177) |
| 2037 | 7.66 (7.11, 8.21) | 5.98 (5.56, 6.41) | 12410 (5116, 19704) | 94045 (40626, 147463) | 180.92 (169.22, 192.62) | 141.35 (132.21, 150.49) | 259681 (86786, 432575) | 1986150 (777547, 3194753) |
| 2038 | 7.78 (7.21, 8.35) | 5.99 (5.55, 6.42) | 13402 (5508, 21296) | 107447 (46134, 168759) | 183.62 (171.47, 195.77) | 141.27 (131.93, 150.62) | 279986 (91827, 468145) | 2266136 (869373, 3662898) |
| 2039 | 7.90 (7.32, 8.49) | 5.99 (5.54, 6.43) | 14415 (5911, 22920) | 121862 (52045, 191679) | 186.31 (173.73, 198.90) | 141.17 (131.63, 150.70) | 300677 (96966, 504389) | 2566813 (966339, 4167287) |
| 2040 | 8.02 (7.42, 8.63) | 5.99 (5.54, 6.44) | 15448 (6324, 24572) | 137310 (58369, 216251) | 189.01 (176.00, 202.01) | 141.04 (131.33, 150.74) | 321744 (102209, 541279) | 2888557 (1068548, 4708566) |
| 2041 | 8.15 (7.52, 8.77) | 5.99 (5.53, 6.45) | 16501 (6748, 26253) | 153811 (65118, 242504) | 191.70 (178.29, 205.12) | 140.88 (131.02, 150.74) | 343175 (107563, 578787) | 3231732 (1176111, 5287353) |
| 2042 | 8.27 (7.62, 8.91) | 5.98 (5.52, 6.45) | 17572 (7183, 27962) | 171383 (72301, 270466) | 194.40 (180.59, 208.21) | 140.70 (130.71, 150.70) | 364959 (113031, 616888) | 3596692 (1289142, 5904241) |
| 2043 | 8.39 (7.73, 9.05) | 5.98 (5.51, 6.45) | 18662 (7628, 29697) | 190046 (79929, 300163) | 197.09 (182.90, 211.29) | 140.50 (130.38, 150.62) | 387087 (118616, 655558) | 3983779 (1407758, 6559799) |
| 2044 | 8.51 (7.83, 9.19) | 5.98 (5.50, 6.45) | 19770 (8083, 31457) | 209816 (88012, 331620) | 199.79 (185.22, 214.36) | 140.28 (130.05, 150.50) | 409548 (124321, 694774) | 4393326 (1532079, 7254574) |
| 2045 | 8.63 (7.94, 9.33) | 5.97 (5.49, 6.45) | 20896 (8549, 33242) | 230712 (96561, 364862) | 202.49 (187.55, 217.42) | 140.03 (129.70, 150.36) | 432332 (130147, 734516) | 4825658 (1662226, 7989090) |

Supplementary Figure 1: Age-specific rates of diet-attributable CMD burden by sex in the United States.

Supplementary Figure 2: The age-standardized death rates of (A) cardiovascular diseases; (B) diabetes; (C) chronic kidney disease attributable to dietary risks in the US, by sex, 1990-2023

Supplementary Figure 3: The estimated annual percent changes of (A) cardiovascular diseases; (B) diabetes; (C)chronic kidney disease from 1990 to 2023 by age groups

Supplementary Figure 4: Associations with SDI and diet-attributable CVD burden across all US states (Spearman correlation: -0.749 for ASMR; -0.712 for ASDR)

Supplementary Figure 5: Associations with SDI and diet-attributable diabetes burden across all US states (Spearman correlation: -0.768 for ASMR; -0.751 for ASDR)

Supplementary Figure 6: Associations with SDI and diet-attributable diabetes burden across all US states (Spearman correlation: -0.689 for ASMR; -0.691 for ASDR)

Supplementary Figure 7: The estimated annual percent changes of (A) age-standardized death rate; (B) age-standardized DALY rate of cardiovascular diseases attributable to dietary risks across United States.

Supplementary Figure 8: The estimated annual percent changes of (A) age-standardized death rate; (B) age-standardized DALY rate of diabetes attributable to dietary risks across United States.

Supplementary Figure 9: The estimated annual percent changes of (A) age-standardized death rate; (B) age-standardized DALY rate of chronic kidney disease attributable to dietary risks across United States.

Supplementary Figure 10: Leading dietary risks of cardiometabolic diseases across the United States. (A: DALYs of cardiometabolic diseases; B: Deaths of cardiometabolic diseases)

Supplementary Figure 11: The age-period-cohort model of the DALYs of cardiovascular diseases attributable to dietary risks in the United States, 1990-2023

Supplementary Figure 12: The age-period-cohort model of the DALYs of diabetes attributable to dietary risks in the United States, 1990-2023

Supplementary Figure 13: The age-period-cohort model of the DALYs of chronic kidney diseases attributable to dietary risks in the United States, 1990-2023

Supplementary Figure 14: (A) The projected age-standardized death rate of cardiovascular diseases by 2045; (B) The averted deaths of cardiovascular diseases from 2024-2045.

Supplementary Figure 15: (A) The projected age-standardized death rate of diabetes by 2045; (B) The averted deaths of diabetes from 2024-2045.

Supplementary Figure 16: (A) The projected age-standardized death rate of chronic kidney disease by 2045; (B) The averted deaths of chronic kidney disease from 2024-2045.
